# Computational Analysis of Cytokine Release Following Bispecific T-Cell Engager Therapy: Applications of a Logic-Based Model

**DOI:** 10.3389/fonc.2022.818641

**Published:** 2022-03-08

**Authors:** Gianluca Selvaggio, Silvia Parolo, Pranami Bora, Lorena Leonardelli, John Harrold, Khamir Mehta, Dan A. Rock, Luca Marchetti

**Affiliations:** ^1^ Fondazione The Microsoft Research - University of Trento Centre for Computational and Systems Biology (COSBI), Rovereto, Italy; ^2^ Pharmacokinetics and Drug Metabolism, Amgen Inc., South San Francisco, CA, United States; ^3^ Quantitative Pharmacology & Disposition, Seattle Genetics, San Francisco, CA, United States; ^4^ Clinical Pharmacology, Modeling and Simulation, Amgen Inc., South San Francisco, CA, United States; ^5^ ADME and Discovery Toxicology, Merck, San Francisco, CA, United States; ^6^ Department of Cellular, Computational and Integrative Biology (CIBIO), University of Trento, Trento, Italy

**Keywords:** QSP modeling, logical modeling, cancer immunotherapy, cytokine release syndrome, CRS

## Abstract

Bispecific T-cell engaging therapies harness the immune system to elicit an effective anticancer response. Modulating the immune activation avoiding potential adverse effects such as cytokine release syndrome (CRS) is a critical aspect to realizing the full potential of this therapy. The use of suitable exogenous intervention strategies to mitigate the CRS risk without compromising the antitumoral capability of bispecific antibody treatment is crucial. To this end, computational approaches can be instrumental to systematically exploring the effects of combining bispecific antibodies with CRS intervention strategies. Here, we employ a logical model to describe the action of bispecific antibodies and the complex interplay of various immune system components and use it to perform simulation experiments to improve the understanding of the factors affecting CRS. We performed a sensitivity analysis to identify the comedications that could ameliorate CRS without impairing tumor clearance. Our results agree with publicly available experimental data suggesting anti-TNF and anti-IL6 as possible co-treatments. Furthermore, we suggest anti-IFNγ as a suitable candidate for clinical studies.

## Introduction

Cancer immunotherapy, by stimulating the immune response against tumor cells, has shown potential for the treatment of numerous cancers. Several components of the immune system have been targeted to elicit an effective anticancer response, ranging from immune cells to cytokines and immune checkpoints (1). Among them, T-cells, given their key role in inducing antigen-specific cytotoxicity, have been the focus of several immunotherapies (2).

Chimeric antigen receptor T (CAR-T) cells and bispecific T-cell engaging antibody molecules (BiAbs), in particular, have been designed to boost the recruitment of T-cells and induce tumor cell lysis (3, 4). CAR-T cells are patient-derived T-cells, genetically engineered to recognize a cancer antigen through a chimeric antigen receptor (CAR) expressed on the cell membrane. BiAbs are made of two single-chain variable fragments (scFv) joined by a flexible linker. One scFv binds to the invariant CD3 part of the T-cell receptor, while the second binds to a tumor-associated antigen. Thus, it physically links a T-cell to a tumor cell, inducing T-cell activation. This, in turn, leads to the release of cytolytic proteins and tumor cell lysis (4–6). Compared to CAR-T cells, BiAbs manufacturing does not require the *ex vivo* genetic manipulation of autologous T-cells for their activation, but instead it employs endogenous T-cells and redirects their action to achieve efficacy (3, 4). The first approved BiAbs therapy was blinatumomab (Blincyto^®^), an antibody specific for the B cell marker CD19 and the T-cell receptor CD3 (7, 8), which received approval for the treatment of B cell acute lymphoblastic leukemia by the United States Food and Drug Administration (FDA) in 2014 (9).

A critical concern regarding the use of both CAR-T cells and BiAbs therapies is the overactivation of the immune response leading to an adverse effect called cytokine release syndrome (CRS). CRS is a systemic inflammatory response characterized by pronounced release of cytokines (10, 11). CRS symptoms vary across patients, from flu-like mild symptoms (mild CRS) to potentially life-threatening adverse events, such as hypotension and hypoxia (severe CRS) (12, 13).

CRS onset is thought to be caused by the activation of T-cells and bystander immune cells such as monocytes/macrophages by these therapies, leading to the release of cytokines and chemokines that mediate a systemic inflammatory response. Interleukin-6 (IL-6) has been recognized to have a key role in CRS pathogenesis, and clinical treatments with tocilizumab (anti-IL-6 monoclonal antibody) have shown efficacy to control CRS symptoms in patients (14–16). Other cytokines including IL-1, IL-2, IFN-γ, TNFα, GM-CSF, MIP1α, MCP1, IL-5, IL-8, and IL-10 were also found to rise during the course of CRS (17–19), highlighting a complex interplay between cells and cytokines. Despite the identification of these CRS markers, the molecular mechanisms leading to it are not fully understood, thus limiting the development of an effective strategy to manage and possibly prevent CRS onset.

Mathematical models can contribute to better understanding the mechanisms involved in CRS and supporting mitigation strategies, including the design and development of safe and efficacious drugs as well as optimal drug regimens. However, the lack of quantitative information and the complex cytokine network considerably restrict the development of useful predictive models. Chen et al. proposed a semi-mechanistic pharmacokinetic/pharmacodynamic model of BiAbs treatment to simulate the cytokine profile and identify *in silico* candidate dosage regimens to mitigate CRS toxicity (11, 20, 21). IL-6 was used as a proxy of the inflammatory response, and an immunomodulatory feedback loop affecting its secretion was used to mimic the priming effect. Analogously, Hosseini et al. (22) developed a mechanistic quantitative systems pharmacology (QSP) model describing B-cell and T-cell dynamics in the presence of bispecific antibodies (e.g., mosunetuzumab and blinatumomab). Hosseini’s model describes IL-6 dynamics as a function of T-cell abundance and activation state and, as Chen et al. (11), explores step dosing as a strategy to reduce cytokine peak levels.

Other QSP models investigated the impact of disease burden and dosage regimen on cytokine levels in the context of CAR-T cell therapy. In the model of Hardiansyah et al. (23), IL-6, IL-10, and IFN-γ are produced by CAR-T-cells as a function of their abundance and a phenomenological function of IL-10 inhibits IFN-γ production. Hopkins et al., instead, focused on 9 cytokines and their cross-regulation with a model developed from clinical data related to the anti-CD28 monoclonal antibody TGN1412 (24). The CRS was fitted to a phenomenological function, and a sensitivity analysis was used to assess the most effective cytokine inhibition protocol.

The models developed so far to describe the CRS phenomenon are centered either on the cellular or on the cytokine dynamics, thus neglecting the interplay between the two. In the present work, we used published clinical trials and human cell line data of the biAbs/CAR-T treatment effect (18, 25) and experimental measurements of cytokines in humanized mice (26) to support the development of a logical model of biAbs immunotherapy, with a focus on CRS development. The logical formalism was selected to properly describe a heterogeneous network, composed of cells and cytokines, given the availability of semiquantitative data and lack of a proper mechanistic description of the phenomenon. The resulting model was used to evaluate key elements and identify the best strategy to mitigate CRS without affecting the tumor killing capacity of T-cells.

## Materials and Methods

### Model Construction and Simulation

**Figure 1:**
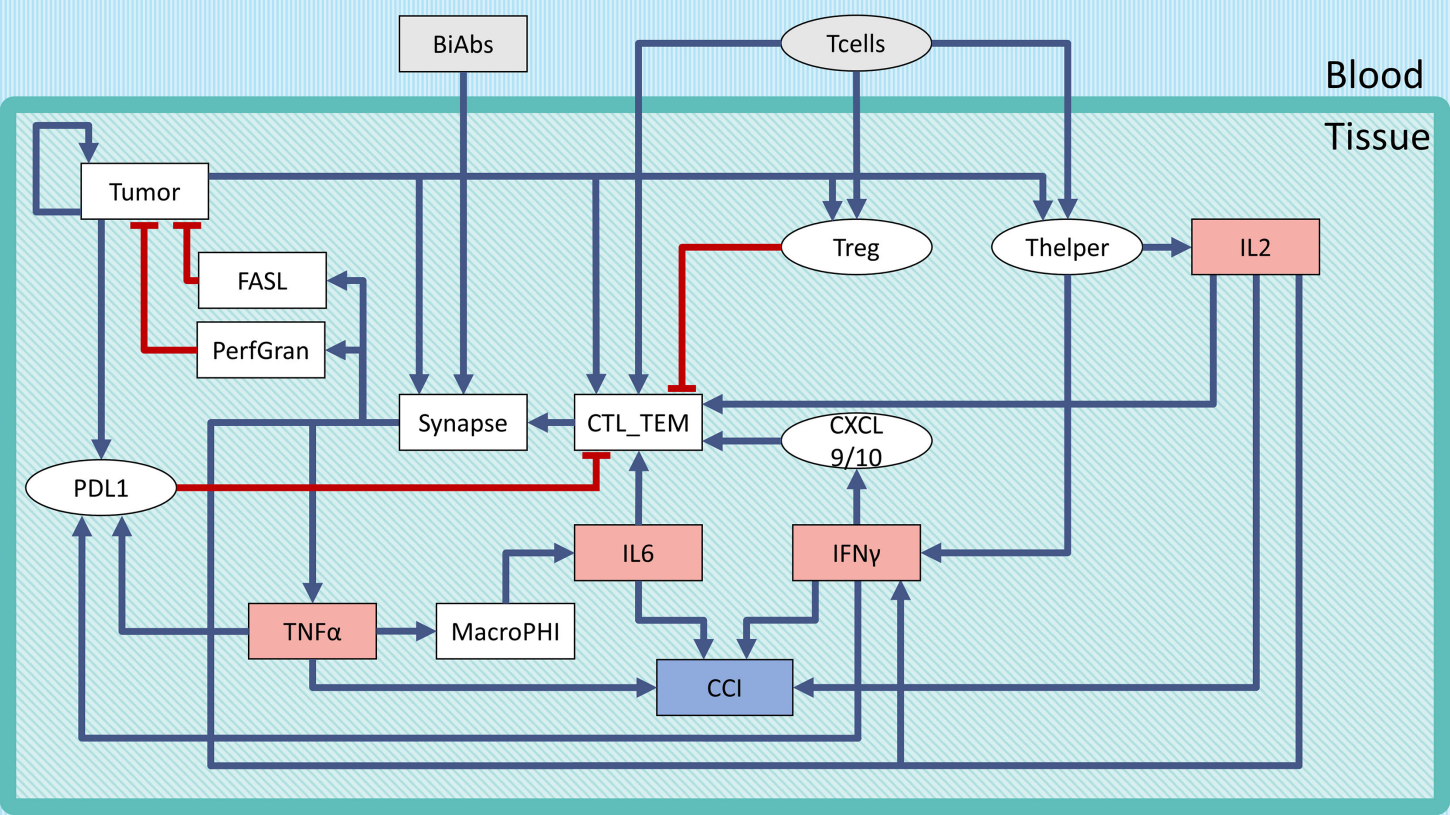
Regulatory network of CRS following BiAbs treatment. Inputs are denoted in gray, cytokines in light red, and the output in light purple-filled boxes. Inhibitions are indicated with red blunt arrows, activations by blue arrows. Rectangular-shaped boxes identify multivalued components, while circular ones are associated with Boolean variables. The CCI output node represents the Combined Cytokine Index (CCI), a proxy readout of systemic CRS.

The logical model describing the bispecific antibody mechanism of action (MoA) and CRS onset (**Figure 1**) was built using GINsim (27) (version 3.0.0b, http://ginsim.org), a tool dedicated to the logical formalism, evaluating the stable states of the model and offering several convenient features for extended analysis.

Considering the stochastic nature of asynchronous update, when simulating the logical model dynamically, each stable state (attractor) had an associated reachability probability, which quantified the trajectories leading to that attractor. The reachability probability was calculated using the built-in GINsim functionality, which implements the Firefront algorithm (28).

A more quantitative description of the dynamics was obtained using the software MaBoSS (29, 30) (https://maboss.curie.fr). MaBoSS computes stochastic trajectories and provides the time evolution of probabilities of the component values. In classical logic formalism, time is not a continuous process but is rather represented by discrete steps (an iteration counter) linking events sequentially. In MaBoSS, a node is associated with a logical rule and a transition rate. The model is casted as a continuous time Markov process, using the Gillespie algorithm to simulate the network.

In our model, we did not make assumptions on the system dynamics, thus all rates were equal to 1. To simulate, we used the following setup: time step of 0.01, 10^5^ runs, and a maximum time of 200 [a.u.]. The transition rate sensitivity analysis simulated the system with the same parameters as above, but with a 10 times increase (or decrease) in activation (or deactivation) rate for each variable.

### Sensitivity Calculation

To calculate the sensitivity of cytokine release or the tumor with respect to a specific alteration (mutation) of the states and/or transition probabilities, we applied the following formula:


$$S_{mutX}^{Y}=\frac{\left(AUC{\left(Y(t)\right)}^{mutX}-AUC{\left(Y(t)\right)}^{WT}\right)}{AUC{\left(Y(t)\right)}^{WT}}$$

where AUC(Y) indicates the area under the curve of the variable Y, mutX is the mutation under consideration, and WT is the original model or wild type. We selected the AUC as metric to integrate in a unique index the change in the shape due to a shift in time or a lowering of the peak value.

We considered two types of mutations for the node analysis:

- Knockdown (KD): we set an upper bound of the component, by limiting the highest value it could reach. The mutation was defined as KDXX, where XX is a number representing the inhibited fraction (e.g., KD50 implied that the components were constrained to vary between 0 and 0.5 instead of between 0 and 1 in the wild type).
- Overexpression (OE): we set a lower bound of the component, by increasing the lowest value to be held. The mutation was defined as OEXX, where XX is a number representing the increased basal activity (e.g., OE50 implied that the components were constrained to vary between 0.5 and 1 instead of between 0 and 1 in the wild type).

To evaluate the sensitivity of the transition rates, we mutated the model by either increasing or decreasing by 10 times the selected transition rate.

## Results

### Model Diagram

To describe the tumor microenvironment and the BiAbs MoA, we built a minimal regulatory network that incorporates the current biological knowledge (**Figure 1**). We identified from the literature the major variables influencing the activation of cytokine release and tumor cell elimination. The rules describing the variables’ behavior (see **Supplementary Information Table S1**) and the regulatory interactions were defined based on experimental evidence (see **Supplementary Information Table S2**). In **Supplementary Information Table S3**, we reported the range of cytokine concentrations in blood found in the literature (18).

We defined two inputs (gray boxes in **Figure 1**): BiAbs and T-cells. The former represents the drug amount administered to the patient, with 3 levels (no drug—0, medium dose—0.5, and high dose—1). The latter indicates the presence/absence of circulating T-cells that can be recruited at the tumor site (T-cell extravasation). BiAbs, upon reaching the neoplastic tissue, link cytotoxic T-cells (CTL) cells to tumor cells forming an immune synapse. The activated immune cells will then secrete perforin and granzyme and expose on their membrane the transmembrane protein FASL. The variable and the logic rules describing these processes are encoded in the corresponding nodes. The tumor node is a multivalued variable (0, 0.5, and 1) describing the three levels of tumor burden (absent, medium, and heavy). Among the various cytokines involved in the immune response, we selected IL-6, IL-2, TNFα, and IFN-γ based on their importance in CRS, as documented in the scientific literature (15, 16, 18, 26, 31) and in the experimental data (see **Supplementary Information Figure S5**).

Since the literature highlighted a dose–effect relationship between BiAbs and cytokine release (32), we encoded this dependency in TNFα with 3 levels (0, 0.5, and 1). Among the TNFα-mediated regulations, we included the recruitment of macrophages, which have been shown to be the main responsible for the release of IL-6 (16), and the induction of the immune checkpoint and exhaustion marker PDL1 by cancer cells (together with IFN-γ).

Other cytokines, such as IL-2 and IFN-γ, were encoded with a 4-level variable (0, 0.33, 0.66, and 1). The low value, 0.33, represented the common abundance in the physiological setting, while the two high values (0.66 and 1) were achievable only on T-cell activation due to bispecific antibody-T-cell synapse complex formation.

The severity of the inflammation was described by a multivalued output, Combined Cytokine Index (CCI), with 5 levels (0–4) that corresponded to the number of cytokines at their maximum level in the system. This output serves as a local proxy for the systemic CRS.

### Effect of Bispecific T-Cell Engaging Antibody Therapy

#### Asymptotic Analysis

The asymptotic behavior of the model was evaluated by computing its stable states (SS). The model reaches 10 fixed points with no periodic steady states (**Table 1**) that can be summarized as 3 patterns: SS1 representing a system without tumor, SS2 showing an established tumor without infiltration of immune cells (cold tumor), and SS3 representing an *in situ* carcinoma with an ongoing immune response (hot tumor) (33).

**Table 1:** Stable states of the logical model.

Stable states	BiAbs	T-cells	Tumor_Cell	T_reg	T_helper	CTL_TEM	Synapse	PerfGran	FASL	PDL1	CXCL9_10	MacroPHI	IL6	IL2	IFNg	TNFa	CCI
**SS1**	*	*	**0**	**0**	**0**	**0**	**0**	**0**	**0**	**0**	**0**	**0**	**0**	**0**	**0**	**0**	**0**
**SS2**	*	**0**	**1**	**0**	**0**	**0**	**0**	**0**	**0**	**0**	**0**	**0**	**0**	**0**	**0**	**0**	**0**
**SS3**	**0**	**1**	**1**	**1**	**1**	**0.5**	**0**	**0**	**0**	**0**	**0**	**0**	**0**	**0.33**	**0.33**	**0**	**0**

The values’ color (gradient from red to blue) denotes the level of the node: 0, 0.33, 0.5, 1. The wild card * (gray asterisk) indicates any admissible level for that node. SS1 and SS2 identify patterns by grouping together different stable states.

To understand the reachability properties of the stable states, we simulated the evolution of the model starting from an initial state with Tumor low (0.5) without BiAbs treatment, Synapse, FASL, and PerfGran, while leaving all the other variables free to assume any admissible value. All simulations reached the fixed point SS3, thus showing that the system when untreated evolves toward a condition of fully grown tumor, immune cell infiltration, and limited cytokine release.

The fixed point SS3 was used as initial state to perturb with BiAbs treatments. The considered doses were capable of depleting asymptotically the tumor without residual cytokines, ending in a state that belongs to the SS1 pattern (see **Supplementary Information**—Wild type model dynamics). However, given the transitory nature of the CRS phenomenon and the necessity to interrupt the treatment whenever the cytokines become harmful to the patients, we further investigated the model behavior by assessing how the two doses differed in terms of dynamics.

#### Dynamic Analysis

**Figure 2:**
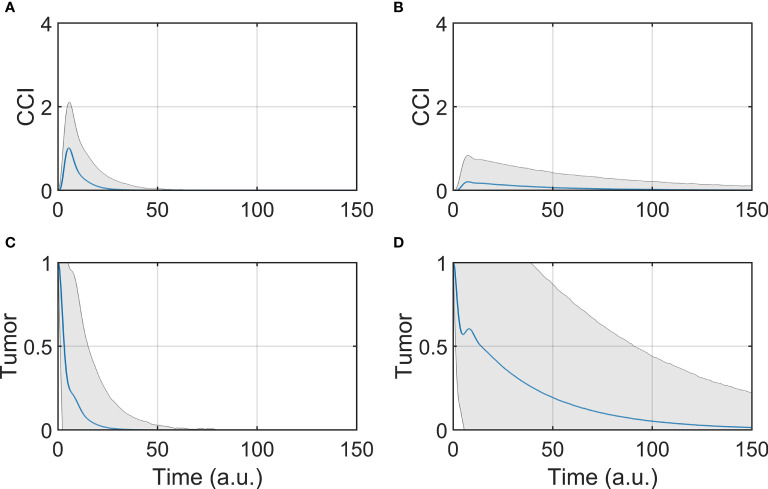
Tumor and CCI response. **(A, C)** Show respectively the CCI and Tumor response if the system is treated with a high dose (BiAbs = 1), **(B, D)** show instead the CCI and Tumor response with a low dose (BiAbs = 0.5). The blue line represents the mean, while the shaded area is the standard deviation.

The dynamic response was studied by simulating the evolution of the model components overtime, after initializing the system to the tumor state (SS3). The BiAbs dose was maintained constant through the simulation. In **Figure 2** are reported the average responses of the model when treated with either low or high doses.

The two doses achieved the final behavior shown in Asymptotic analysis described above. However, the tumor (**Figures 2C, D** **)** was cleared at different rates in a dose-dependent fashion, and with a wider dispersion for low doses. The dynamics of the single cytokine (see **Supplementary Information**—Comparison with experimental data) showed a dose response to the drug and a qualitative agreement with the experimental data, also in terms of relative timing of the peak values (IL-6 delayed when compared with IL-2 of IFN-γ).

A trade-off between therapy effectiveness and safety emerged from this analysis, highlighted here by CCI dynamics (**Figures 2A, B** **)**. At low dose, CCI showed a small peak with a long tail, while high dose induced a sharp and high peak response. It can be presumed that the lower the levels of cytokine release, the lower the risk of clinical CRS and hence the safer the treatment. In order to identify possible co-treatments to administer with BiAbs, we systematically calculated the sensitivities of Tumor and CCI with respect to perturbation of the model components.

### Sensitivity Analysis

The results presented in **Figure 2** highlight that a lower dose of a BiAbs would ameliorate the CCI but at the cost of a slower tumor clearance. We explored this trade-off between safety (cytokine levels) and efficacy (eradication of tumor) for a high-dose setting by conducting a systematic sensitivity analysis. In the following sections, we refer to the wild-type model (WT) as the scenario in which the unmodified network of **Figure 1** is treated with BiAbs.

#### Mutations: Knockdown and Overexpression

The importance of each model component was assessed by perturbating its value and evaluating the relative effect of its mutation on the Tumor clearance and the CCI dynamics, under treatment with high dose of BiAbs.

The results for Tumor and CCI sensitivities were combined and ranked, based on their sum (**Table 2**). This highlighted the most valuable targets to reduce CCI and at the same time preserve the tumor clearance. Given the spike-like nature of CCI, upon high dose of BiAbs (see **Figure 2A**), we also reported the Severe CCI-Risk, a variable representing the probability for an individual subject to reach at least CCI-3.

**Table 2:** Sensitivities of CCI and tumor with respect to model perturbations.

Rank	Perturbation	Tumor Sensitivity [%]	CCI Sensitivity[%]	Severe CCI-Risk[%]
1	**FASL-OE100**	-71.1	-69.9	4.8
2	**PerfGran-OE100**	-71.1	-69.9	4.9
3	**IFNg-KD66**	-29.0	-42.2	0.0
4	**IFNg-KD100**	-28.6	-41.6	0.0
5	**PerfGran-OE50**	-38.7	-18.2	9.9
6	**FASL-OE50**	-38.2	-18.4	9.9
7	**TNFa-KD100**	-2.7	-44.1	0.0
8	**TNFa-KD50**	-2.0	-43.7	0.0
9	**IFNg-KD33**	-1.2	-25.9	4.5
10	**IL2-KD66**	0.1	-26.2	4.7
11	**IL2-KD33**	0.2	-26.1	4.7
12	**T-reg-KD100**	-35.5	13.2	21.9
13	**CTL-TEM-OE100**	-35.5	13.2	22.0
14	**CTL-TEM-OE50**	-35.6	13.6	22.1
15	**PDL1-KD100**	-35.6	14.1	22.0
16	**IL6-KD50**	0.0	-17.8	9.5
17	**IL6-KD100**	0.1	-17.5	9.4
18	**MacroPHI-KD50**	0.2	-17.6	9.5
19	**MacroPHI-KD100**	0.4	-17.8	9.5
20	**CXCL9-10-OE100**	-12.4	3.1	14.5
21	**T-helper-OE100**	-0.6	-0.4	11.8
22	**IFNg-OE33**	-0.2	-0.4	12.0
23	**IL2-OE33**	0.3	-0.5	11.7
**24**	**WT**	**0.0**	**0.0**	**12.0**
25	**T-reg-OE100**	0.1	0.1	11.9
26	**IL6-OE50**	0.0	1.4	12.5
27	**CXCL9-10-KD100**	3.2	-1.4	11.1
28	**CTL-TEM-KD50**	3.7	-1.6	11.2
29	**MacroPHI-OE50**	0.0	3.8	12.7
30	**IL2-OE66**	-0.1	6.1	12.9
31	**TNFa-OE50**	1.9	9.0	13.3
32	**PerfGran-KD50**	29.5	28.0	13.7
33	**FASL-KD50**	30.0	27.8	13.7
34	**FASL-KD100**	44.3	33.6	14.1
35	**PerfGran-KD100**	44.5	33.6	14.3
36	**CTL-TEM-KD100**	>>100	-100.0	0.0
37	**Synapse-KD100**	>>100	-100.0	0.0
38	**Synapse-KD50**	>>100	-100.0	0.0
39	**PDL1-OE100**	>>100	-86.9	1.9
40	**IL2-KD100**	>>100	-82.6	1.2
41	**IFNg-OE66**	>>100	-58.7	7.4
42	**Synapse-OE100**	-58.5	>>100	100.0
43	**T-helper-KD100**	>>100	-44.4	8.0
44	**Synapse-OE50**	>>100	-27.1	13.5
45	**MacroPHI-OE100**	0.1	>>100	21.9
46	**IL2-OE100**	0.2	>>100	20.3
47	**IL6-OE100**	0.3	>>100	26.6
48	**IFNg-OE100**	>>100	>>100	11.8
49	**TNFa-OE100**	>>100	>>100	12.8

The different mutations are ranked by the sum of the effects on the Tumor and CCI; blue values indicate an improvement, while red values are a worsening (the values are percentages relative to the wild-type model). The third column (Severe CCI-Risk) indicates the probability to reach at least CRS-3. The sensitivities that were reported to be >>100 indicate that the Tumor (or CCI) value for that mutant was not zero at the end of the simulation.

The first two rows of **Table 2** clearly suggests that an overexpression of granzyme and perforin or FASL could lead to a fast tumor clearance with minimal cytokine production. These are, however, simulation scenarios and their feasibility in experimental and clinical setting needs to be evaluated.

The model suggests that IFN-γ may be an optimal target; indeed, both a partial (KD66) inhibition and a complete (KD100) inhibition induce a significant decrease of the CCI levels without affecting the antitumoral effect. The reduction in CCI was noticeable even when the knockdown was weak (KD33), although in this case the synergistic effect with the tumor killing was negligible.

The ranked sensitivities in **Table 2** also highlighted that targeting TNF-α or IL-6 had an effect on CCI, reducing the risk index from 12% to 0% and ~9%, respectively, plus reducing in general the exposure to the cytokines (in terms of relative area under the curve, see *Sensitivity Calculation*). These results are in agreement with literature findings, as anti-TNF-α or anti-IL6 therapies have been employed in clinical practice to reduce CRS (26), albeit with varying degrees of success.

Interestingly, perturbation in some variables showed dual behaviors (e.g., PDL1-KD100 or CXCL9/10-OE100). The inhibition of PDL1 showed a beneficial effect on the tumor reduction, as its ability to inactivate CTL cells had dropped. However, this was counterbalanced by a significant increase in the CCI and CCI-Risk, respectively +14% and +22% with respect to the wild type. The explanation for this effect may be in the extreme efficacy of the therapy with no mechanisms of controlling the synapse formation and, thus, the cytokines’ release. Analogously, the overexpression of CXCL9/10 enhanced the recruitment of CTL cells, which showed a beneficial effect on the antitumoral capability at the expense of an increase in the CCI-risk.

#### Transition Rates: Slow and Fast

Although the network perturbations shown in the previous paragraph were fundamental to the understanding of the key components of the system, the effects on the component dynamics also needed to be addressed for a complete analysis of the system. We perturbed the rates of activation (Up-Rate) or deactivation (Down-Rate) of each variable.

We computed the perturbations’ effect on the Tumor and CCI variables and reported the resulting sensitivities in **Tables 3**, **4** (the complete process can be found in the **Supplementary Information** – Sensitivities to update rate perturbations).

**Table 3:** Sensitivities of CCI and tumor with respect to perturbation rates, where each rate was decreased 10 times compared to the wild type case.

Rank	Perturbation	Tumor Sensitivity[%]	CCI Sensitivity[%]	Severe CCI-Risk [%]
1	**u-IFNg**	-24.1	-35.3	2.0
2	**u-TNFa**	-2.1	-41.4	0.8
3	**d-FASL**	-16.7	-9.5	11.9
4	**d-PerfGran**	-16.5	-9.6	11.9
5	**u-IL2**	0.3	-24.4	5.1
6	**d-CTL-TEM**	-30.0	10.6	20.0
7	**u-PDL1**	-30.0	10.9	19.9
8	**u-IL6**	-0.3	-16.5	9.4
9	**u-MacroPHI**	0.0	-16.4	9.4
10	**u-Tumor-Cell**	-9.4	-2.3	11.9
11	**d-CXCL9-10**	-2.2	0.4	11.8
12	**u-T-helper**	-0.2	-0.4	11.8
13	**u-T-reg**	-0.4	-0.1	11.8
14	**d-T-reg**	0.3	-0.4	11.9
**15**	**WT**	**0.0**	**0.0**	**12.0**
16	**d-T-helper**	0.0	0.1	11.9
17	**u-CXCL9-10**	2.2	-1.1	11.3
18	**d-IL6**	-0.2	48.8	11.9
19	**d-MacroPHI**	0.3	61.7	12.0
20	**u-PerfGran**	39.1	30.3	14.2
21	**d-PDL1**	69.7	-0.3	11.6
22	**u-FASL**	39.6	30.8	13.8
23	**u-CTL-TEM**	77.4	-0.7	11.3
24	**d-IL2**	0.0	85.0	13.6
25	**d-IFNg**	92.8	89.4	14.0
26	**u-Synapse**	>>100	-10.4	1.2
27	**d-Synapse**	-5.5	>>100	54.5
28	**d-TNFa**	23.5	100.0	14.6
29	**d-Tumor-Cell**	>>100	>>100	20.9

The different mutations are ranked by the sum of the effects on the Tumor and CCI; blue values indicate an improvement, while red values are a worsening (the values are percentages relative to the wild type model). For every variable, we differentiated between the rate of activation, or up-rate (u-X), and the rate of deactivation, or down-rate (d-X). The third column (Severe CCI-Risk) indicates the probability to reach at least CCI-3. The sensitivities that were reported to be >>100 indicate that the Tumor (or CCI) value for that mutant was not zero at the end of the simulation.

**Table 4:** Sensitivities of CCI and Tumor with respect to perturbation rates, where each rate was increased 10 times compared to the wild-type case.

Rank	Perturbation	Tumor Sensitivity[%]	CCI Sensitivity[%]	Severe CCI-Risk[%]
1	**d-Tumor-Cell**	-38.3	-41.2	6.2
2	**u-PerfGran**	-25.1	-25.3	9.1
3	**u-FASL**	-25.2	-25.0	9.1
4	**u-Synapse**	-38.4	3.2	15.8
5	**d-IFNg**	-7.5	-11.9	9.8
6	**d-TNFa**	-0.5	-15.6	9.6
7	**d-Synapse**	19.3	-35.2	4.3
8	**d-IL2**	-0.2	-9.9	10.0
9	**u-CTL-TEM**	-10.3	0.3	12.7
10	**d-PDL1**	-7.1	-0.7	12.0
11	**d-MacroPHI**	0.2	-5.3	11.6
12	**d-IL6**	0.1	-4.2	11.8
13	**u-CXCL9-10**	-3.7	0.5	12.5
14	**d-T-reg**	-0.5	-0.5	11.9
15	**u-T-reg**	-0.1	-0.7	11.8
16	**d-CXCL9-10**	0.0	-0.4	11.9
**17**	**WT**	**0.0**	**0.0**	**12.0**
18	**u-T-helper**	0.2	0.0	12.0
19	**d-T-helper**	0.5	-0.1	11.9
20	**u-Tumor-Cell**	2.7	0.0	11.8
21	**u-IL6**	0.4	6.2	13.8
22	**u-MacroPHI**	0.1	8.8	13.9
23	**d-FASL**	9.4	4.6	11.8
24	**d-PerfGran**	9.3	5.3	11.8
25	**u-IL2**	0.4	19.0	14.9
26	**u-PDL1**	38.0	-7.6	7.1
27	**d-CTL-TEM**	39.0	-8.3	7.0
28	**u-TNFa**	7.1	26.2	15.3
29	**u-IFNg**	28.0	21.9	12.4

The different mutations are ranked by the sum of the effects on the Tumor and CCI; blue values indicate an improvement, while red values are a worsening (the values are percentages relative to the wild-type model). For every variable, we differentiated between the rate of activation, or up-rate (u-X), and the rate of deactivation, or down-rate (d-X). The third column (Severe CCI-Risk) indicates the probability to reach at least CCI-3. The sensitivities that were reported to be >>100 indicate that the Tumor (or CCI) value for that mutant was not zero at the end of the simulation.

The systematic reduction of the system rates (**Table 3**) showed similar effects to those observed in the perturbation results (**Table 2**). A reduction in the rate of production of IFN-γ was the most desirable outcome as it synergistically increased the antitumoral capability and reduced the CCI. Interestingly, mutations that would maintain the synapse active for a longer time (as decreasing the rate of PDL1 production or of the Synapse inactivation) had a critical effect on the risk of the patient to develop CCI. Analogously, we calculated the sensitivities for CRS and Tumor when the rates were increased (**Table 4**).

Similarly to what we showed for overexpression, increasing the rate of activation of granzyme and perforins or FASL had a positive effect on tumor clearance and CCI reduction (**Table 4**). Interestingly, increasing the rate of degradation of IFN-γ was still an effective choice, but suboptimal with respect to decreasing the cytokine production rate.

### Delayed Treatments

**Figure 3:**
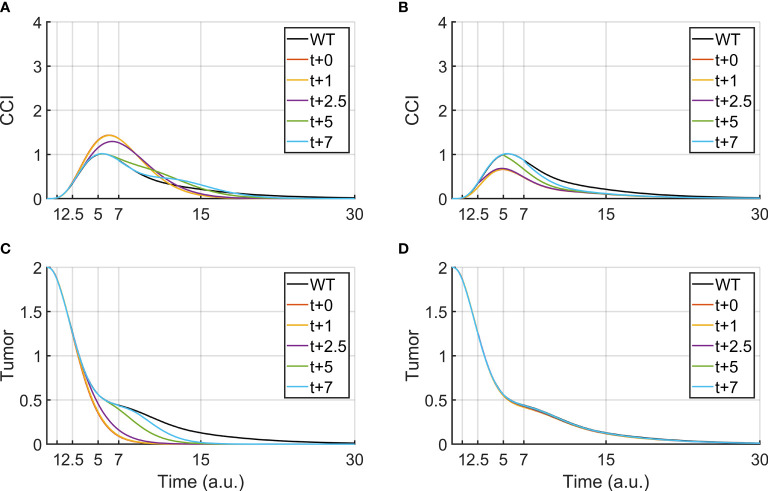
Tumor clearance and CCI response after a continuous BiAbs treatment (high dose) starting at t = 0 (WT condition). The different lines represent Tumor and CCI trends when BiAbs therapy is provided at time t = 0 and either anti-PDL1 (KD100, **A, C**) or anti-TNFα (KD100, **B, D**) is administered at time t = +1, +2.5, +5, and + 7 [a.u.].

In clinical practice, other drugs can be administered together with BiAbs therapy to increase the tumor clearance or reduce the CRS. Therefore, it is crucial to understanding how to leverage combined therapies, by identifying the administration-time window that provides the best outcome. Although our logical model lacks quantitative time, it was used as a supporting tool to investigate the effects of a delayed administration of the secondary drug. To this purpose, we focused on anti-PDL1/anti-PD1 and anti-TNFα, two treatments that are currently used in the clinical practice. **Figures 3A, C** report the effect of anti-PDL1/anti-PD1 treatments (PDL1-KD100) administered after the bispecific antibody. We observed that an early treatment induces a faster clearance of the tumor and an acute CRS response, which can be mitigated by postponing the anti-PDL1 drug, even though this translated in a slower tumor clearance.

Anti-TNFα therapies (TNFa-KD100, **Figures 3B, D** **)**, on the other hand, showed almost no improvement of tumor clearance but a drastic reduction of CCI if co-administered early after BiAbs. Similar results have been obtained for TNFα-KD50 (see **Supplementary Information** – Anti- TNFα therapy).

## Discussion

A computational model based on the logic describing the key elements of tumor microenvironment and the mechanism of action of the bispecific T-cell engaging antibody is presented here. The modeling framework we applied can circumvent the typical challenges of parameter estimation and model qualification that make the development of continuous systems pharmacology models challenging. The choice of a logical formalism also helps to include modules which lack detailed kinetic information but have multitude of qualitative information coming from diverse sources and contexts, making it easier to quickly integrate all available information and evaluate diverse hypothesis in a robust fashion.

Previous work focused on either the cellular response or the cytokine response. For example, Hosseini et al. (22) focused on the cellular dynamics and used a single-cytokine (IL-6) proxy for cytokine release syndrome, while Hopkins et al. (34) generated a model of CRS relying solely on the cytokines profile. The proposed modeling framework stands out in terms of how it handles the cross talk between the cellular and cytokine components.

Our model simulations confirm the potential of the bispecific T-cell engaging antibody to effectively eliminate tumor, given sufficient dose to overcome the tumor growth. The effect of dose levels in our model is manifested as a difference in the dynamic behavior of the system, including the cytokine release and the rate of tumor elimination. The simulations show a monotonic decrease in tumor elimination rates with high BiAbs doses with a reduction of the effectiveness toward the end of treatment (change in slope). Conversely, lower BiAbs doses presented a non-monotonic curve of tumor inhibition, which, after a first drastic drop, increases followed by a slow decay. The two different dose levels showed contrasting behaviors in terms of cytokine release as well, as demonstrated by the dynamics of the CCI. The simulations show a sharp and high CCI for high BiAbs doses and a smaller long-tailed peak for low doses.

In the simulation scenarios presented in this work, we considered a constant infusion of BiAbs; thus, the decrease in the cytokine’s response can be attributed to the tumor clearance. Model simulations using knockdown or overexpression of nodes showed that the current model predictions agree with published work on the effect of anti-IL-6 and anti-TNF-α interventions as strategies to counteract cytokine release. Importantly, our model shows that anti-IL-6 and anti-TNF-α therapies combined with a high dose of BiAbs were capable of reducing the CRS risk as measured by CCI, without impairing the antitumoral drug effect (26). Interestingly, a modest decrease in anti-IFN-γ (KD66) not only reduced the cytokine release but also increased the tumor clearance. Notably, also enhancing the recruitment of CTL cells *in situ* (e.g., inducing CXCL9/10) was effective in inducing the tumor clearance but gave an unfavorable outcome for the cytokine syndrome. Mutating the rates of activation and deactivation of each node identified IFN-γ as the optimal target, suggesting that inhibition of the cytokine’s production rate would have a more meaningful effect than increasing its clearance. Our analysis thus suggests that acting on IFN-γ may be a good strategy to control CRS risk in patients.

The systematic mutation studies performed in sensitivity analysis showed the combined therapies that could ameliorate the BiAbs treatment in terms of CRS reduction and tumor clearance. For the development of an effective combined therapy, it is critical to identify the optimal time window to administer the secondary drug to optimize the tumor clearance along with reduction on CRS risk. We conducted simulations using anti-PDL1 (35) and anti-TNFα (26). The former showed a trade-off with the major improvements on tumor clearance obtained by an early co-administration with BiAbs, while a reduction in CRS risk was possible only with a delay in the therapy combination, suggesting a late optimal time window to mitigate the CCI while maintaining an acceptable tumor clearance. On the contrary, an early co-administration of anti-TNFα had the most striking effect, with stronger effects the sooner the therapy was administered.

The current work showed how logical QSP models can be used to investigate complex phenomena and generate new testable hypothesis. The inclusion of a more fine-grained description of the pathways governing the cytokine release and the immune activation will make the model closer to *in vivo* conditions, thus further increasing its predictive power. The rapidly emerging biological and clinical data across diverse T-cell-activating therapies can be leveraged to build a more robust and representative model. Methodologically, this work made no assumptions on the reaction’s priority, meaning that within the model components there was no hierarchy for updating: a deeper knowledge of the biology can be used to set classes of update during the simulation and, thus, refine the dynamics we obtained (and indirectly the sensitivities). Another step toward mapping the modeling time to the realistic time can be obtained by properly setting the model parameters, in the dynamic simulation, to match experimental data (30) or recast the system as logic-based ODE model (36). Despite the limitation, the approach described here shows an efficient and effective way to quickly evaluate the currently available hypothesis and generate new ones. For example, we showcased the utility of the model in uncovering and evaluating newer targets for CRS-risk minimization and how to better administrate existing ones.

## Data Availability Statement

The full set of model simulations and analysis are available in the article/**Supplementary Material**, together with the model rules and annotations.

## Author Contributions

GS developed the computational model and performed the analysis under the supervision of LM and JH. SP, PB, and LL reviewed the literature and identified the biological mechanisms to be included in the computational model. DR and KM provided project support in Amgen. LM provided overall guidance of the project. All authors contributed to the article and approved the submitted version.

## Funding

The authors declare that this study received funding from Amgen. The funder was involved in the study design, collection, analysis, interpretation of data, the writing of this article and the decision to submit it for publication.

## Conflict of Interest

KM is a current employee of Amgen. JH and DR were employees of Amgen while this study was conducted. DR is currently employed by Merck; JH is employed by Seattle Genetics. GS, SP, PB, LL, and LM were contracted by Amgen while this research was conducted, while no commercial or financial relationship is in existence with Merck or Seattle Genetics.

The remaining authors declare that the research was conducted in the absence of any commercial or financial relationships that could be construed as a potential conflict of interest.

The authors declare that this study received funding from Amgen. The funder was involved in the study design, collection, analysis, interpretation of data, the writing of this article and the decision to submit it for publication.

## Publisher’s Note

All claims expressed in this article are solely those of the authors and do not necessarily represent those of their affiliated organizations, or those of the publisher, the editors and the reviewers. Any product that may be evaluated in this article, or claim that may be made by its manufacturer, is not guaranteed or endorsed by the publisher.
