# Supplementary material for: Computational Analysis of Cytokine Release Following Bispecific T-Cell Engager Therapy: Applications of a Logic-Based Model
Source: Front Oncol. 2022 Mar 8;12:818641. doi: 10.3389/fonc.2022.818641 (PMC8957948; doi:10.3389/fonc.2022.818641)
Supplement: Supplementary file 1 [file DataSheet_1.pdf]

Computational analysis of cytokine release following bispecific T-cell engager therapy: Applications of a logic-based model.

Gianluca Selvaggio, Silvia Parolo, Pranami Bora, Lorena Leonardelli, John Harrold, Khamir Mehta, Dan Rock, Luca Marchetti

Supplementary Information

Contents

Model nodes annotations 3
Model interactions annotations 6
Cytokine concentrations in CRS patients 7
Wild type model dynamics 8
  High BiAbs dose 8
  Low BiAbs dose 9
Sensitivity calculation 9
Variable perturbations effect on tumor clearance 10
  Over-Expression 10
  Knock-Down 11
  Tumor clearance sensitivities to variable perturbations 12
Variable perturbations effect on CRS 13
  Over-Expression 13
  Knock-Down 14
  CRS sensitivities to variable perturbations 15
Update rate perturbation effects on tumor clearance 16
  Up-Rate 16
    Fast 16
    Slow 17
  Down-Rate 18
    Fast 18
    Slow 19
  Tumor sensitivities to update rates perturbations 20
Update rate perturbation effects on CRS 20
  Up-Rate 21
    Fast 21

Slow 22
Down-Rate 23
Fast 23
Slow 24
CRS sensitivities to update rate perturbations 24
Delayed combined therapy analysis 25
Anti-PDL1 therapy 25
Anti-TNFα therapy 27
TNFα-KD50 27
TNFα-KD100 30
References 32

Model nodes annotations

Table S1: Model component annotations and rules

Node	Max value	Annotations	Rules
**BiAbs**	2	**BiAbs**<br><br>0 Absence<br>1 Low Dose<br>2 High Dose	
**blood_Tcell**	1	**circulating T-cells**<br><br>0 Absence<br>1 Circulating cells	
**Tumor_Cell**	2	**Tumor cell**<br><br>0 Absence<br>1 Small tumor<br>2 Large Tumor	**Lvl 1:** Tumor_Cell & (PerfGran:1 | FASL:1) & !FASL:2 & !PerfGran:2<br><br>**Lvl 2:** !(PerfGran | FASL) & Tumor_Cell
**T_reg**	1	**T-regulatory**<br><br>0 Absence<br>1 Recruited and Activated	**Lvl 1:** Tumor_Cell & blood_Tcell
**T_helper**	1	**T-helper**<br><br>0 Absence<br>1 Recruited and Activated	**Lvl 1:** Tumor_Cell & blood_Tcell
**CTL_TEM**	2	**Cytotoxic T lymphocytes**<br><br>0 Absence<br>1 Recruited and Low Activation<br>2 Recruited and High Activation	**Lvl 1:** (Tumor_Cell & blood_Tcell & T_reg & !PDL1 & !(IL6 | IL2) & CXCL9_10)| (Tumor_Cell & blood_Tcell & T_reg & !PDL1 & (IL6 | IL2) & !CXCL9_10)| (Tumor_Cell & blood_Tcell & !T_reg & PDL1 & !(IL6 | IL2) & CXCL9_10)| (Tumor_Cell & blood_Tcell & !T_reg & PDL1 & (IL6 | IL2) & !CXCL9_10)

			**Lvl 2:** (Tumor_Cell & blood_Tcell & !T_reg & !PDL1 & (IL6 | IL2 | CXCL9_10))| (Tumor_Cell & blood_Tcell & T_reg & !PDL1 & (IL6 | IL2) & CXCL9_10)| (Tumor_Cell & blood_Tcell & !T_reg & PDL1 & (IL6 | IL2) & CXCL9_10)
**Synapse**	2	**Synapse complex formed by BiAbs+Tumor+CTL**<br><br>0 Absence<br>1 Mild complex formation<br>2 High complex formation	**Lvl 1:** Tumor_Cell & BiAbs:1 & CTL_TEM:1<br><br>**Lvl 2:** Tumor_Cell & ((BiAbs:2 & CTL_TEM:2) | (BiAbs:2 & CTL_TEM:1) | (BiAbs:1 & CTL_TEM:2))
**PerfGran**	2	**Perforin and Granzyme**<br><br>0 Absence<br>1 Low<br>2 High	**Lvl 1:** Synapse:1<br><br>**Lvl 2:** Synapse:2
**FASL**	2	**Tumor necrosis factor ligand superfamily**<br><br>0 Absence<br>1 Low<br>2 High	**Lvl 1:** Synapse:1<br><br>**Lvl 2:** Synapse:2
**PDL1**	1	**Programmed cell death 1 ligand 1**<br><br>0 Absence<br>1 Expressed	**Lvl 1:** Tumor_Cell & (IFNg:3 | IFNg:2 | TNFa:2)
**IL6**	2	**Interleukin-6**<br><br>0 Absence<br>1 Low<br>2 High	**Lvl 1:** MacroPHI:1<br><br>**Lvl 2:** MacroPHI:2
**IL2**	3	**Interleukin-2**<br><br>0 Absence<br>1 Low<br>2 Medium<br>3 High	**Lvl 1:** T_helper & !Synapse<br><br>**Lvl 2:** Synapse:1<br><br>**Lvl 3:** Synapse:2

**IFNg**	3	**Interferon gamma**<br><br>0 Absence<br>1 Low<br>2 Medium<br>3 High	**Lvl 1:** T_helper & !Synapse<br><br>**Lvl 2:** Synapse:1<br><br>**Lvl 3:** Synapse:2
**TNFa**	2	**Tumor necrosis factor alpha**<br><br>0 Absence<br>1 Low<br>2 High	**Lvl 1:** Synapse:1<br><br>**Lvl 2:** Synapse:2
**CXCL9_10**	1	**C-X-C motif chemokine 9 and C-X-C motif chemokine 10**<br><br>0 Absence<br>1 Presence	**Lvl 1:** IFNg:2 | IFNg:3
**MacroPHI**	2	**Machrophages**<br><br>0 Absence<br>1 Recruited and Low Activation<br>2 Recruited and High Activation	**Lvl 1:** (TNFa:2 | IFNg:2 | IFNg:3) & !(TNFa:2 & (IFNg:2 | IFNg:3))<br><br>**Lvl 2:** (IFNg:2 | IFNg:3) & TNFa:2
**CRS**	4	**Cytokine Release Syndrome**<br><br>Output that account for the number of cytokines that are actively expressed at their maximum level<br><br>0/4 Cytokine<br>1/4 Cytokine<br>2/4 Cytokine<br>3/4 Cytokine<br>4/4 Cytokine	**Lvl 1:** (IL6:2 & !TNFa:2 & !IFNg & !IL2) | (!IL6:2 & TNFa:2 & !IFNg & !IL2) | (!IL6:2 & !TNFa:2 & IFNg & !IL2) | (!IL6:2 & !TNFa:2 & !IFNg & IL2)<br><br>**Lvl 2:** (IL6:2 & TNFa:2 & !IFNg:3 & !IL2:3) | (IL6:2 & !TNFa:2 & IFNg:3 & !IL2:3) | (IL6:2 & !TNFa:2 & !IFNg:3 & IL2:3) | (!IL6:2 & TNFa:2 & IFNg:3 & !IL2:3) | (!IL6:2 & TNFa:2 & !IFNg:3 & IL2:3) | (!IL6:2 & !TNFa:2 & IFNg:3 & IL2:3)<br><br>**Lvl 3:** (IL6:2 & TNFa:2 & IFNg:3 & !IL2:3) | (IL6:2 & TNFa:2 & !IFNg:3 & IL2:3) | (IL6:2 & !TNFa:2 & IFNg:3 & IL2:3) | (!IL6:2 & TNFa:2 & IFNg:3 & IL2:3)<br><br>**Lvl 4:** IL6:2 & TNFa:2 & IFNg:3 & IL2:3

Model interactions annotations

Table S2: Model regulatory interactions and references

Source	Target	Interaction	References
**BiAbs**	**Synapse**	+	(1)
**blood_Tcell**	**CTL_TEM**	+	(2)
**blood_Tcell**	**T_helper**	+	(2)
**blood_Tcell**	**T_reg**	+	(2)
**Tumor_Cell**	**PDL1**	+	(3)
**Tumor_Cell**	**T_helper**	+	(4)
**Tumor_Cell**	**CTL_TEM**	+	(4)
**Tumor_Cell**	**Tumor_Cell**	+	modelling interaction
**Tumor_Cell**	**Synapse**	+	(1)
**Tumor_Cell**	**T_reg**	+	(4)
**T_reg**	**CTL_TEM**	-	(5)
**T_helper**	**IL2**	+	(6)
**T_helper**	**IFNg**	+	(7)
**CTL_TEM**	**Synapse**	+	(1)
**Synapse**	**PerfGran**	+	(8)
**Synapse**	**FASL**	+	(9)
**Synapse**	**TNFa**	+	(1)
**Synapse**	**IL2**	+	(1)
**Synapse**	**IFNg**	+	(1)
**PerfGran**	**Tumor_Cell**	-	(8)
**FASL**	**Tumor_Cell**	-	(9)
**PDL1**	**CTL_TEM**	-	(3)
**IL6**	**CTL_TEM**	+	(10)
**IL6**	**CRS**	+	(11,12)
**IL2**	**CTL_TEM**	+	(13)
**IL2**	**CRS**	+	(12)
**IFNg**	**CXCL9_10**	+	(14)

**IFNg**	**MacroPHI**	+	(7)
**IFNg**	**PDL1**	+	(15)
**IFNg**	**CRS**	+	(11,12)
**TNFa**	**MacroPHI**	+	(16)
**TNFa**	**PDL1**	+	(15)
**TNFa**	**CRS**	+	(11,12)
**CXCL9_10**	**CTL_TEM**	+	(14)
**MacroPHI**	**IL6**	+	(17,18)

Cytokine concentrations in CRS patients

In table are reported the average cytokines values derived from the clinical data of Teachey et al 2016 (19); in which CAR-T cells were used to treat Acute Lymphoblastic Leukemia. The cohort comprised of 50 patients, with the majority (N = 36) developing mild to moderate CRS (grade 0-3) while the remaining (N = 14) developed severe CRS (grade 4-5). The value reported are the peak median value day 1-3. We also report the baseline values for the same patients and for the controls.

Table S3: Cytokines median (range) at day 1-3 peak values (pg/ml) for patients with severe and mild CRS (subjects N=50). Baseline study column reports the values for the same cohort, while baseline control shows the values for normal subjects (N = 10)

	Baseline control	Baseline study	Mild CRS (0-3)	Severe CRS (4-5)
IL6	1.52 (0.29 – 87.6)	7.48 (1.01- 1020)	35.1 (0.38 – 1105)	63.2 (5.29 – 8399)
IL2	1.22 (0.68 – 9.16)	0.83 (0.05 – 1838)	3.71 (0.36 – 1832)	26.5 (0.85 - 138)
IFNγ	19.7 (16.4 – 28.1)	4.58 (0.08 – 22.9)	15.2 (0.13 – 404)	140 (10.5 – 1621)
TNFα	1.32 (0.83 – 1.94)	1.35 (0.02 – 111)	1.68 (0.13 – 105)	1.38 (0.51 – 9.69)

Wild type model dynamics

Below is reported the complete set of variables dynamics for both High and Low dose of BiAbs in the wild type model.

High BiAbs dose

In the figure below are reported the stacked bar charts of the probabilities for all the variables in the case with high dose of BiAbs:

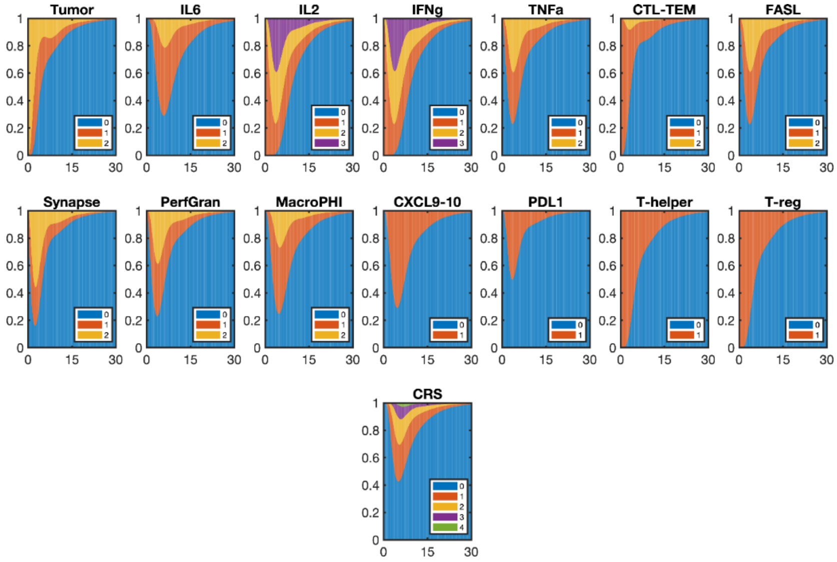

Figure S1: Stacked bar charts, representing for every variable the probabilities that is equal to a specific value in the wild type model.

For every variable we calculate the average profile:

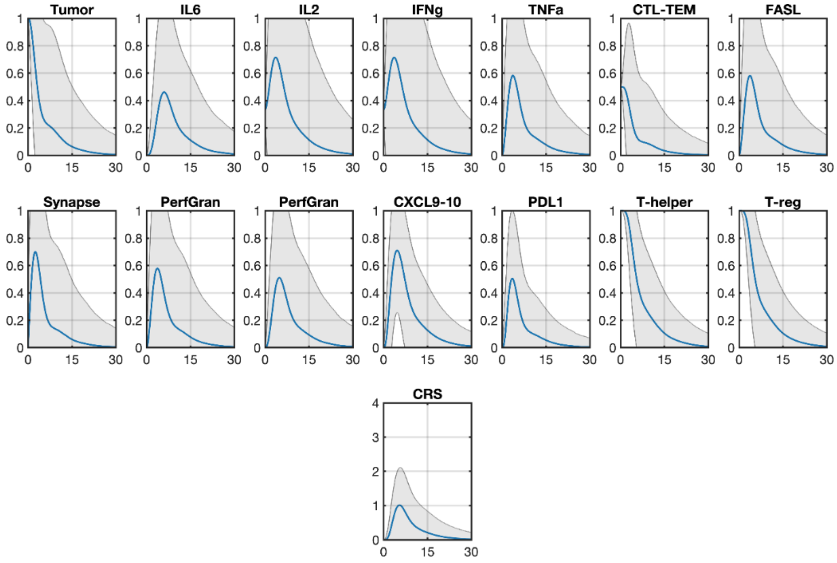

*Figure S2**:** average value of all the variables for the wild type model in low dose of BiAbs conditions. In blue are reported the averages while the shaded grey area shows plus/minus one standard deviation limited to the maximum and minimum values.*

Low BiAbs dose

In the figure below are reported the stacked bar charts of the probabilities for all the variables in the case with low dose of BiAbs:

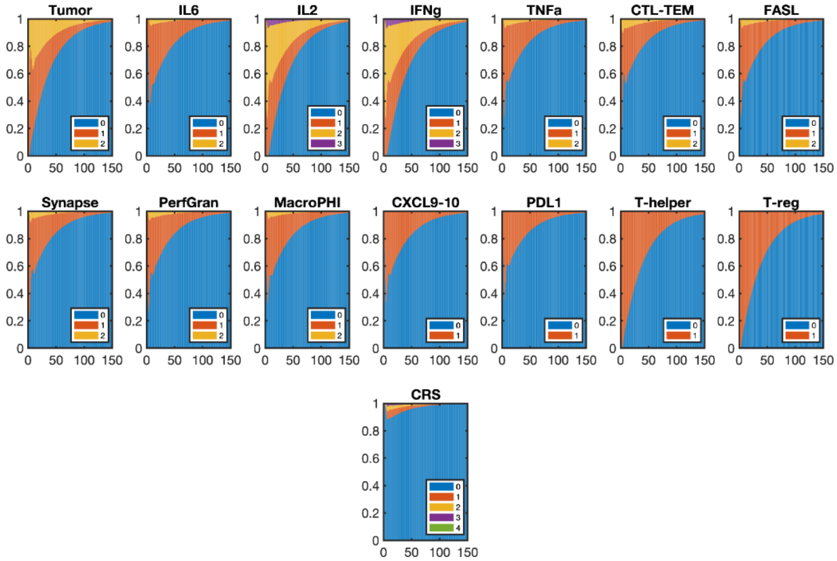

*Figure S3**:** Stacked bar charts, representing for every variable the probabilities that is equal to a specific value in the wild type model.*

For every variable we calculate the average profile:

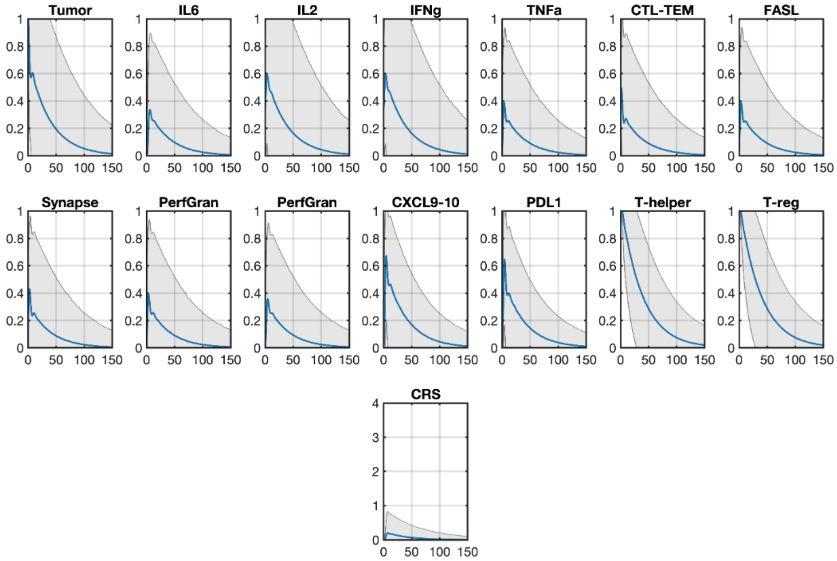

Figure S4: average value of all the variables for the wild type model in low dose of BiAbs conditions. In blue are reported the averages while the shaded grey area shows plus/minus one standard deviation limited to the maximum and minimum values.

Sensitivity calculation

To calculate the sensitivity of CRS or the Tumor with respect to a mutation we applied the following formula:

$$S_{mutX}^{Y} = \frac{(AUC(Y)^{mutX} - AUC(Y)^{WT})}{AUC(Y)^{WT}} \quad (1)$$

where AUC(Y) indicated the area under the curve of the variable (CRS or Tumor), mutX was the considered mutation and WT was the original model or wild type.

Variable perturbations effect on tumor clearance

To assess the effect of model mutations on the Tumor dynamics we simulated the mutated system starting from the final state reported in Figure S4-top, which describes the system state when the tumor is established. The BiAbs input is then set to its maximum and maintained constant for the simulation, as per continuous infusion.

Over-Expression

In each condition we over expressed (OEXX) one single component of the model. The number XX indicates the increased basal activity even in the absence of stimuli (e.g. KD50 the components has a basal level of 0.5, reducing the dynamic range to 0.5-1). The model, then was simulated (#runs=10^5) per each perturbation, the stochasticity of the model and its discrete nature implies that the outcome of a simulation can only provide the probability of that variable to have a certain value. These are plotted in the stacked bar chart below:

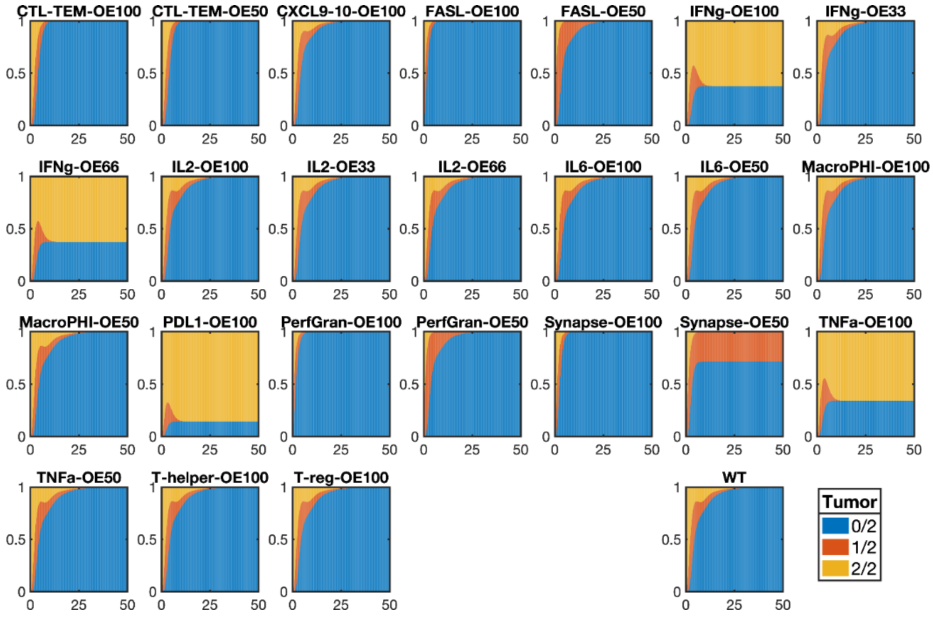

Figure S5: Stacked bar charts, representing the probabilities that the Tumor variable has a specific value at every time step. Each chart represents a single Over-Expression (OE) perturbation.

To obtain the average value of the Tumor node we applied the following formulae:

$$Tumor = \sum_{i=1}^{2} i \cdot P(Tumor = i) \cdot \#runs \quad (2)$$

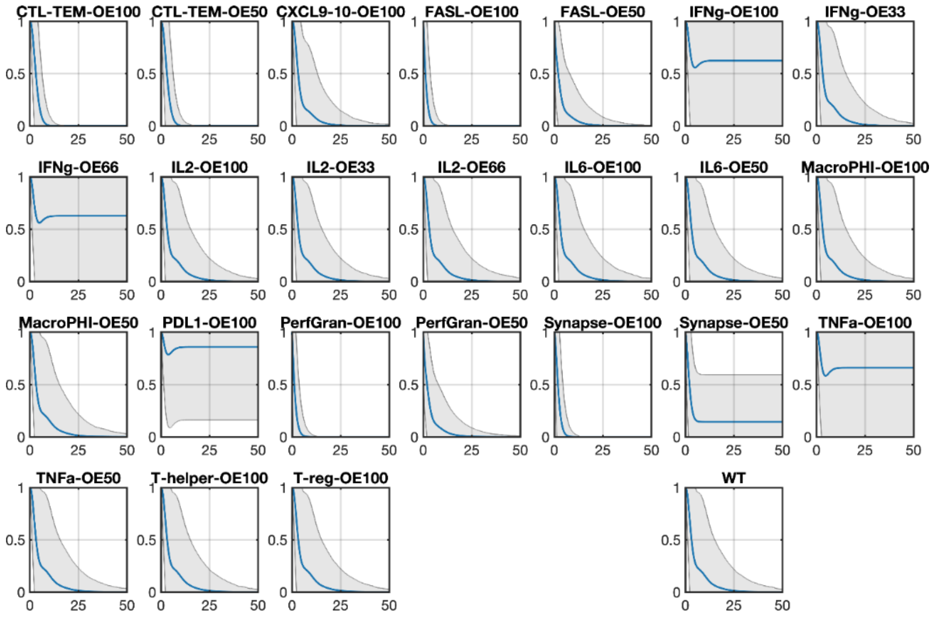

Figure S6: Tumor average value. Each chart represents a single perturbation of the system, in this case Over-Expression. In blue are reported the averages while the shaded grey area shows plus/minus one standard deviation limited to the maximum and minimum values.

Knock-Down

Similarly, as per the Over-Expression mutations we first obtained the probabilities in the different conditions. However, in this case we applied a knock-down mutation KDXX, where XX is the inhibited fraction of the variable (e.g. KD50 the knock-down reduced the dynamic range to 0-0.5)

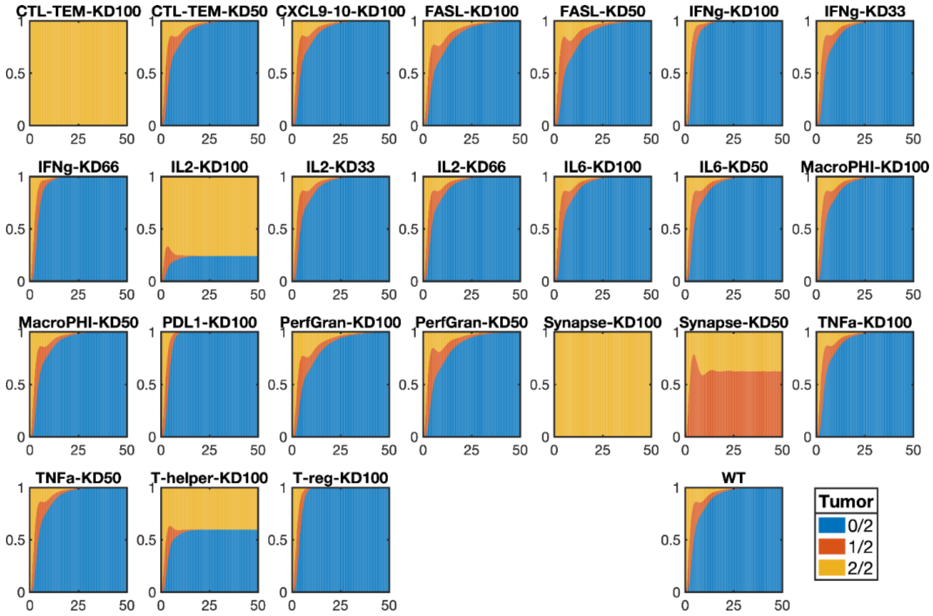

Figure S7: Stacked bar charts, representing the probabilities that the Tumor variable has a specific value at every time step. Each chart represents a single Knock-Down (KD) perturbation.

and then by applying the formula (2) we obtain Figure S8.

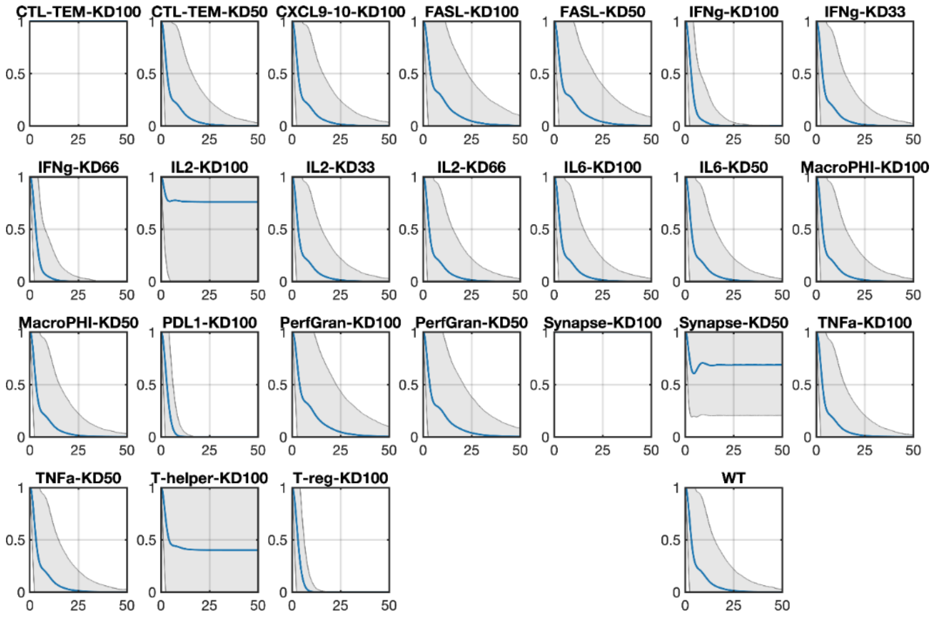

Figure S8: Tumor average value. Each chart represents a single perturbation of the system, in this case Knock-Down. In blue are reported the averages while the shaded grey area shows plus/minus one standard deviation limited to the maximum and minimum values.

Tumor clearance sensitivities to variable perturbations

The average values obtained above are then compared with the wild type behavior (WT), to calculate the sensitivities accordingly to the equation (1).

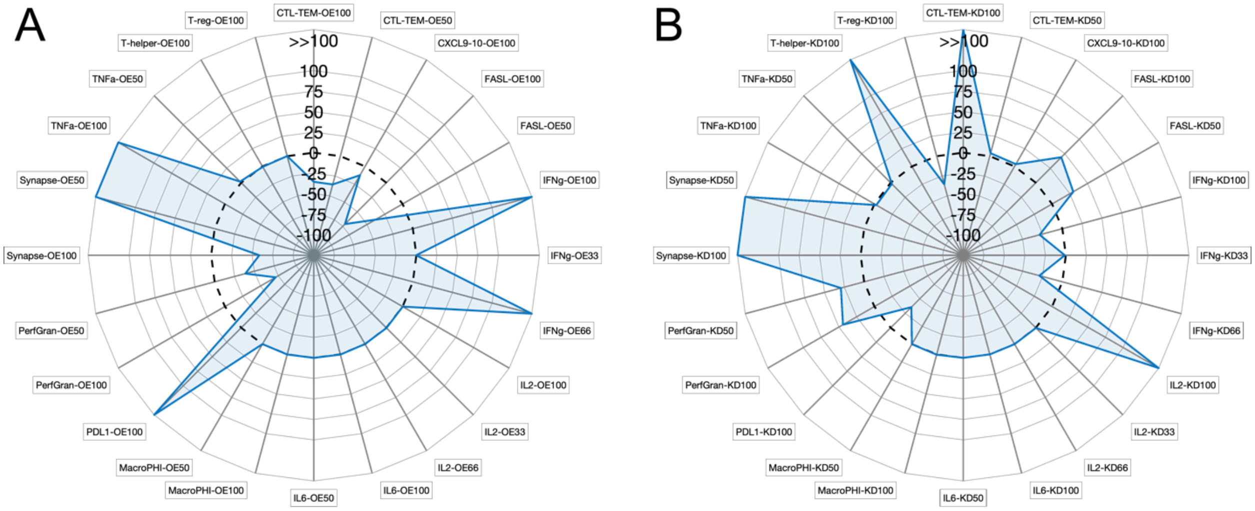

Figure S9: Sensitivity of the Tumor when mutating a node of the network. The mutation can either be knock-down (KDXX) or over-expression (OEXX). The number XX represents for KD the inhiBiAbsd fraction (KD50 means that the dynamic range is 0-0.5) or the increased basal activity for OE (OE50 means that the dynamic range is 0.5-1)

Variable perturbations effect on CRS

To assess the effect of model mutations on the CRS dynamics we simulated the mutated system starting from the final state reported in Figure S4-top, which describes the system state when the tumor is established. The BiAbs input is then set to its maximum and maintained constant for the simulation, as per continuous infusion.

Over-Expression

As per the Tumor mutations, each perturbation was simulated for 10^5 times, and the probability of CRS to have a specific value is reported in the stacked-bar chart below:

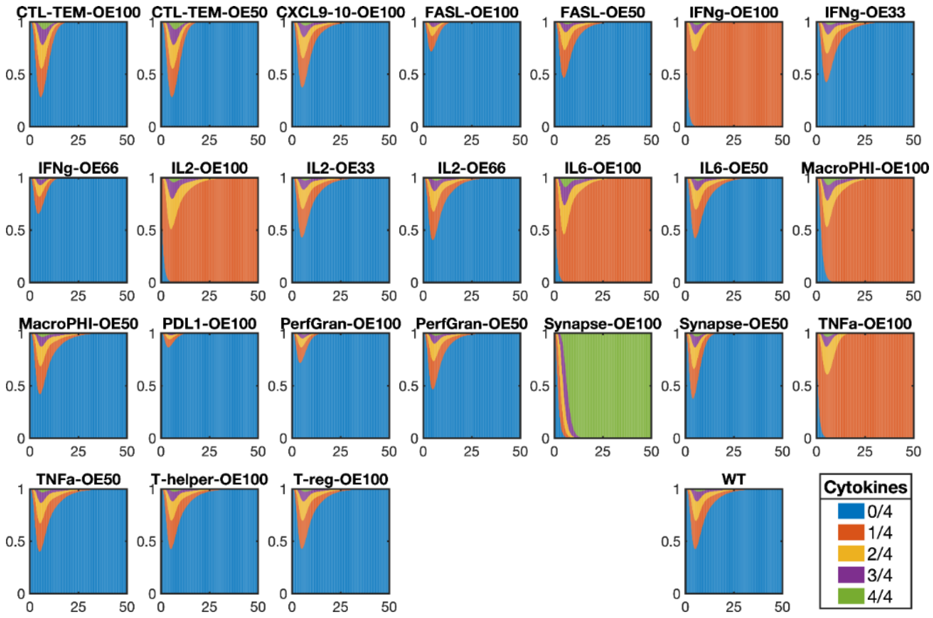

Figure S10: Stacked bar charts, representing the probabilities that the CRS variable has a specific value at every time step. Each chart represents a single Over-Expression (OE) perturbation.

Also, in this case we computed the average of CRS. The value of the variable CRS identifies the number of cytokines that are at their maximum.

$$CRS = \sum_{i=1}^{4} i \cdot P(CRS = i) \cdot \#runs \qquad (3)$$

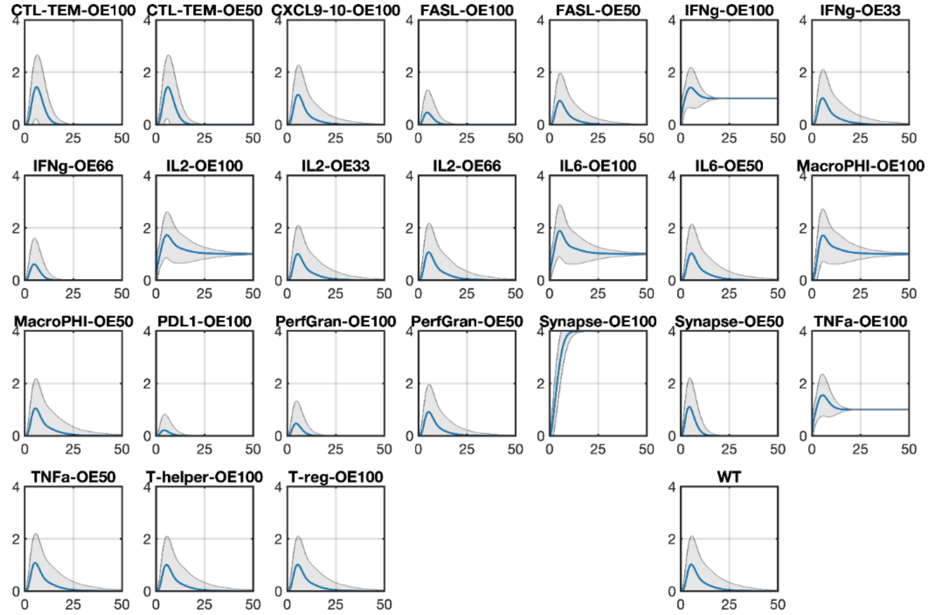

Figure S11: CRS average value. Each chart represents a single perturbation of the system, in this case Over-Expression. In blue are reported the averages while the shaded grey area shows plus/minus one standard deviation limited to the maximum and minimum values.

Knock-Down

Similarly, as per the Over-Expression mutations we first obtained the probabilities in the different conditions. However, in this case we applied a knock-down mutation KDXX, where XX is the inhibited fraction of the variable (e.g. KD50 the knock-down reduced the dynamic range to 0-0.5)

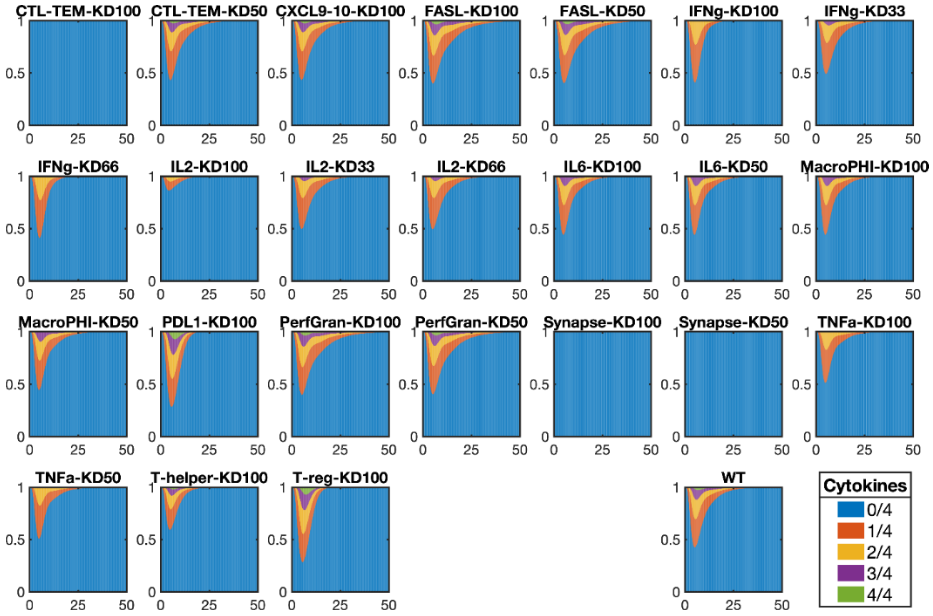

Figure S12: Stacked bar charts, representing the probabilities that the CRS variable has a specific value at every time step. Each chart represents a single Knock-Down (KD) perturbation.

and then by applying formula (3).

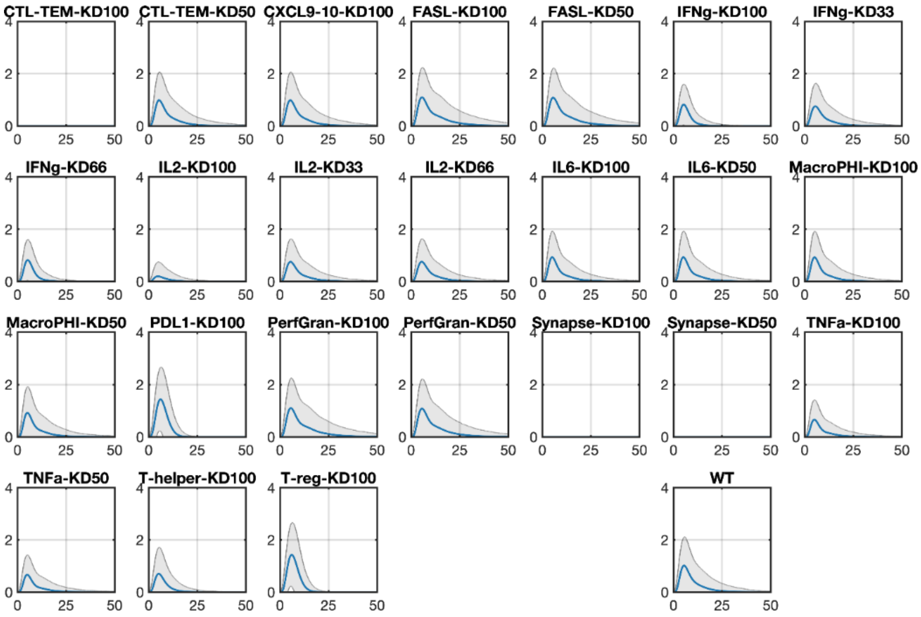

Figure S13: CRS average value. Each chart represents a single perturbation of the system, in this case Knock-Down. In blue are reported the averages while the shaded grey area shows plus/minus one standard deviation limited to the maximum and minimum values.

CRS sensitivities to variable perturbations

The average values obtained above are then compared with the wild type behavior (WT), to calculate the sensitivities accordingly to the equation (1)

.

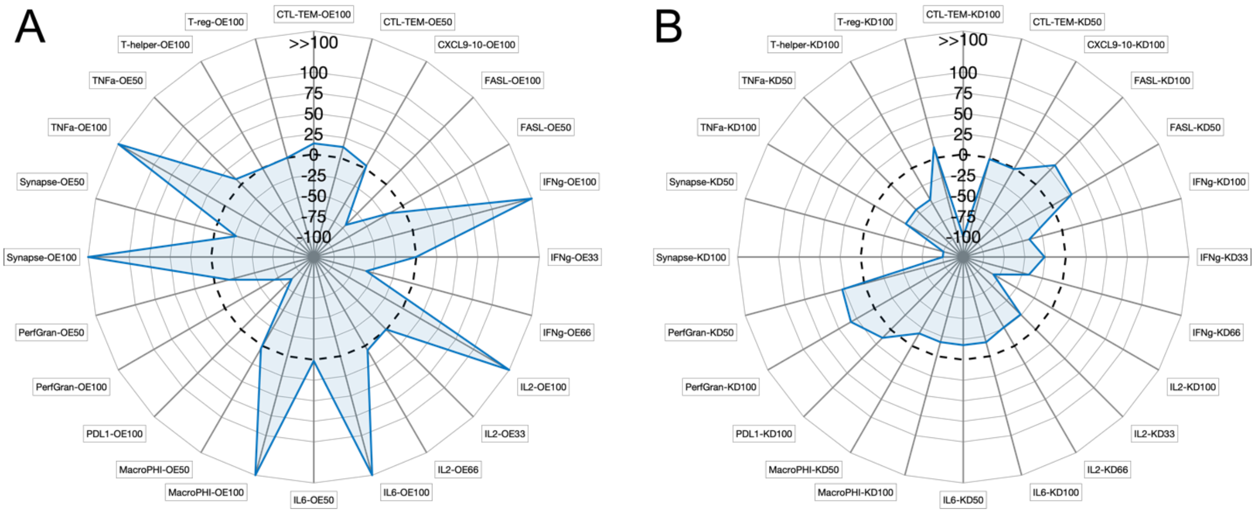

Figure S14: Sensitivity of the CRS when mutating a node of the network. The mutation can either be knock-down (KDXX) or over-expression (OEXX). The number XX represents for KD the inhiBiAbsd fraction (KD50 means that the dynamic range is 0-0.5) or the increased basal activity for OE (OE50 means that the dynamic range is 0.5-1)

Update rate perturbation effects on tumor clearance

To assess the effect of rate mutations on the Tumor dynamics we simulated the modified system starting from the final state reported in Figure S4-top, which describes the system state when the tumor is established. The BiAbs input is then set to its maximum and maintained constant for the simulation, as per continuous infusion.

During stochastic simulations the node to be updated is chosen randomly amongst the others, the rate with which a node is updated is the propensity of a node to be chosen (e.g., gene transcription will have a low rate when compared with kinase phosphorylation). In our model we decided to don't make assumptions on the system dynamics, thus all rates were selected equals.

Up-Rate

We systematically modified the Up-Rate of each variable; these are the propensity of a variable to be selected to vary from 0 1. For each rate we either increased it 10 times (Fast) or decreased it 10 times (Slow).

Fast

In the figure below are reported the stacked-bar charts of the probabilities for the Tumor variable:

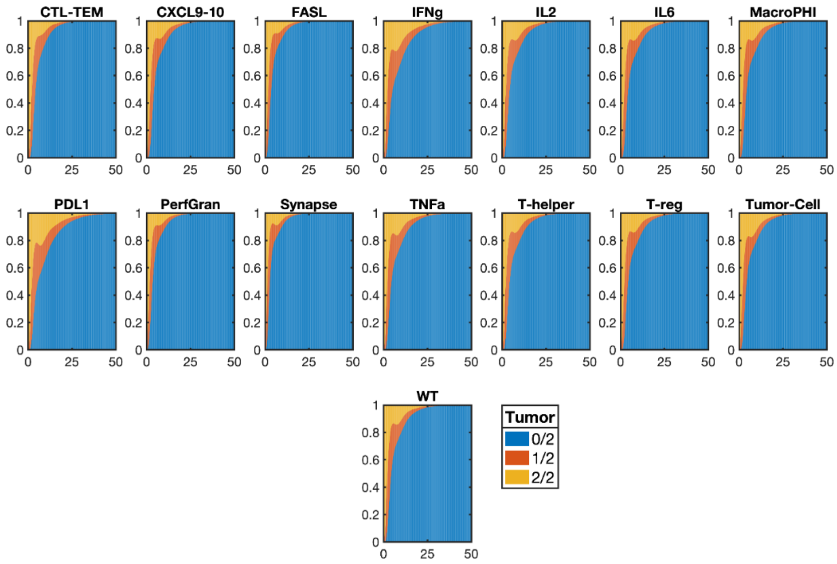

Figure S15: Stacked bar charts, representing the probabilities that the Tumor variable has a specific value at every time step. Each chart represents a single perturbation in which the Up-Rate of the specified variable has been increased 10 times.

and then the average value by applying the formula (2):

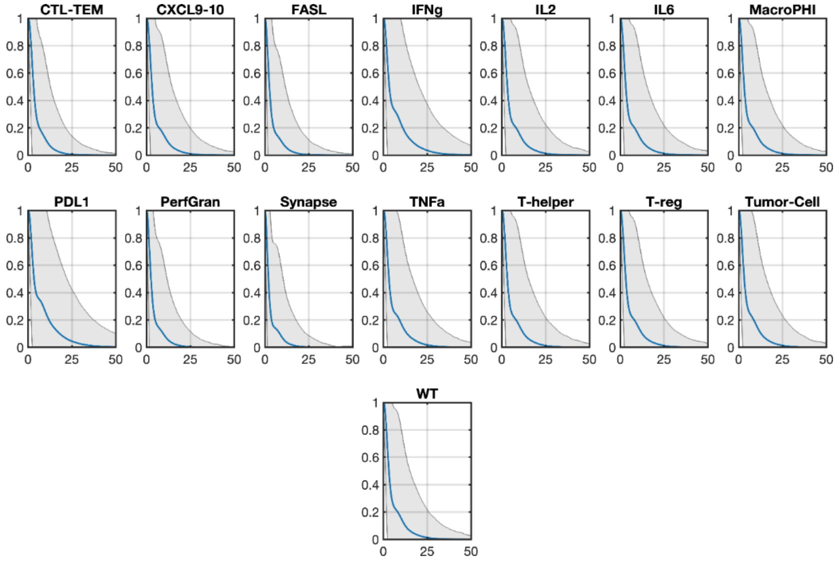

Figure S16: Tumor average value. Each chart represents a single perturbation of the system, in this case increasing 10 times the Up-Rate. In blue are reported the averages while the shaded grey area shows plus/minus one standard deviation limited to the maximum and minimum values.

Slow

In the figure below are reported the stacked-bar charts of the probabilities for the Tumor variable:

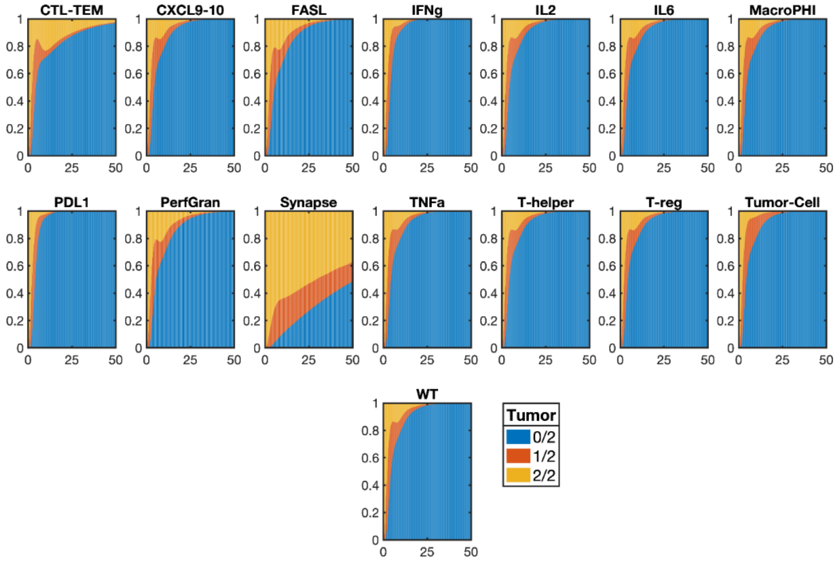

Figure S17: Stacked bar charts, representing the probabilities that the Tumor variable has a specific value at every time step. Each chart represents a single perturbation in which the Up-Rate of the specified variable has been decreased 10 times.

and then the average value by applying the formula (2):

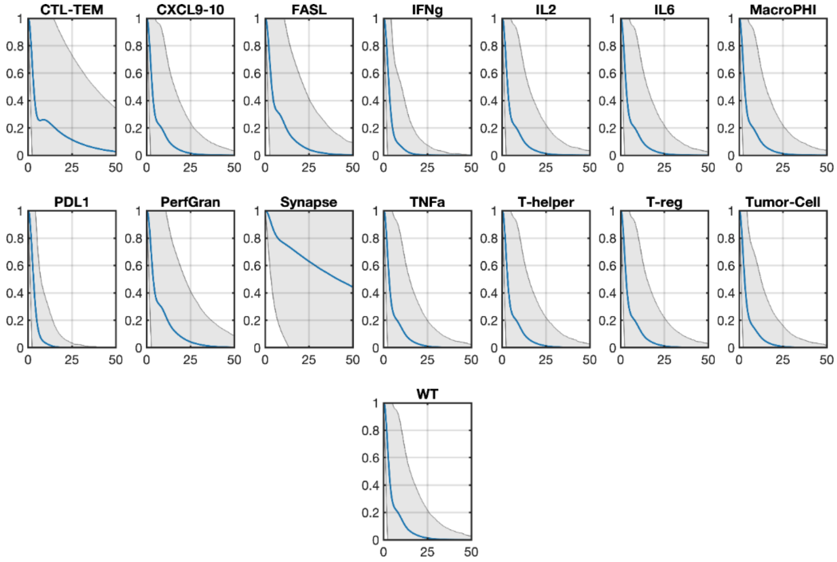

Figure S18: Tumor average value. Each chart represents a single perturbation of the system, in this case decreasing 10 times the Up-Rate. In blue are reported the averages while the shaded grey area shows plus/minus one standard deviation limited to the maximum and minimum values.

Down-Rate

We systematically modified the Down-Rate of each variable; these are the propensity of a variable to be selected to vary from 1 0. For each rate we either increased it 10 times (Fast) or decreased it 10 times (Slow).

Fast

In the figure below are reported the stacked-bar charts of the probabilities for the Tumor variable:

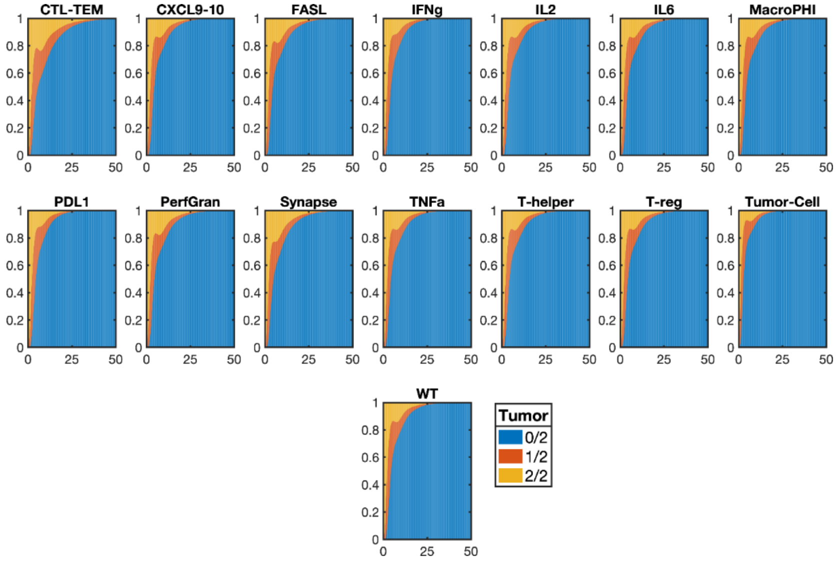

Figure S19: Stacked bar charts, representing the probabilities that the Tumor variable has a specific value at every time step. Each chart represents a single perturbation in which the Up-Rate of the specified variable has been increased 10 times.

and then the average value by applying formula (2)

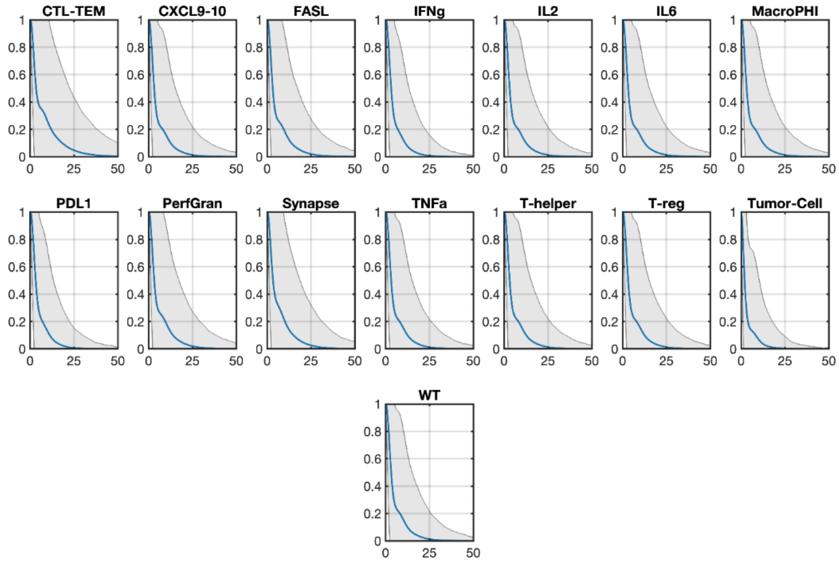

Figure S20: Tumor average value. Each chart represents a single perturbation of the system, in this case increasing 10 times the Down-Rate. In blue are reported the averages while the shaded grey area shows plus/minus one standard deviation limited to the maximum and minimum values.

Slow

In the figure below are reported the stacked-bar charts of the probabilities for the Tumor variable:

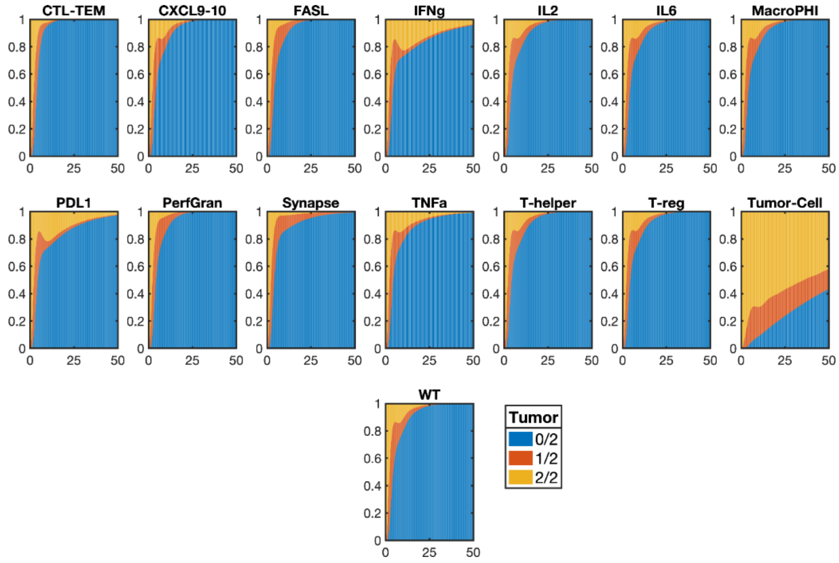

Figure S21: Stacked bar charts, representing the probabilities that the Tumor variable has a specific value at every time step. Each chart represents a single perturbation in which the Up-Rate of the specified variable has been increased 10 times.

and then the average value by applying the formula (2):

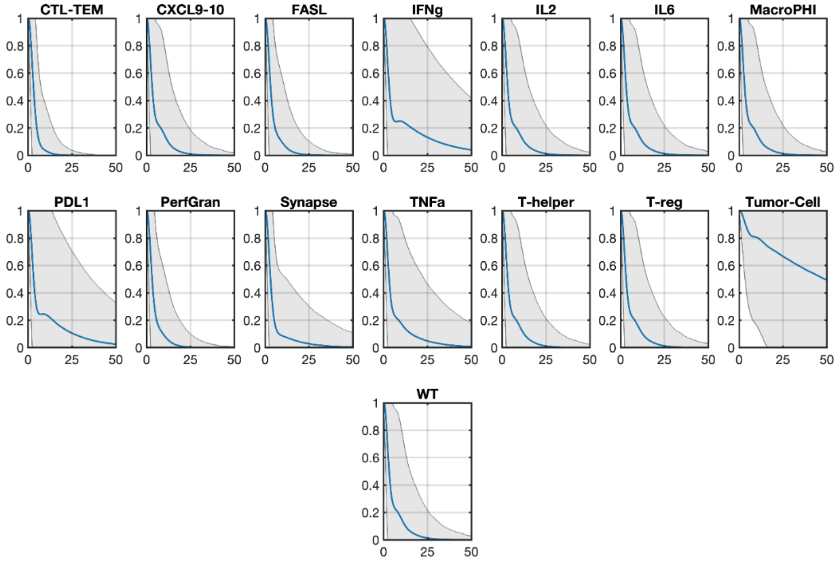

Figure S22: Tumor average value. Each chart represents a single perturbation of the system, in this case decreasing 10 times the Down-Rate. In blue are reported the averages while the shaded grey area shows plus/minus one standard deviation limited to the maximum and minimum values.

Tumor sensitivities to update rates perturbations

The average values obtained are then compared with the wild type behavior, to calculate the sensitivities accordingly to the equation (1).

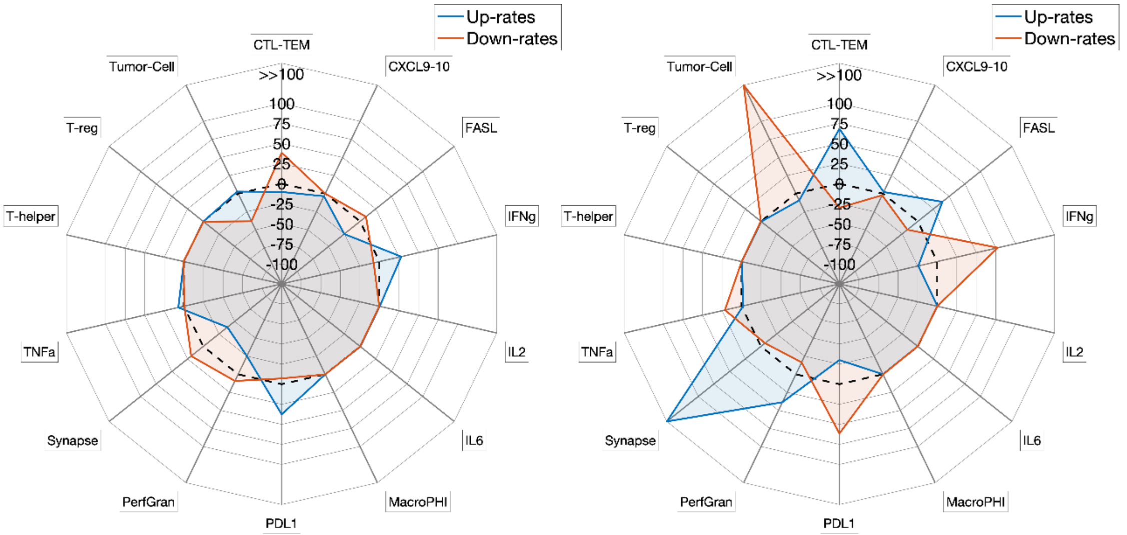

Figure S23:Sensitivity of the Tumor when mutating the rates of increase (Up-rate) or decrease (Down-rate) of a variable. (Right) the rates are decreased 10 times compared to the wild type model, (left) they are increased 10 times.

Update rate perturbation effects on CRS

Similarly, to what has been done to study the effect of the rates mutations on the Tumor clearance we performed the same study on the CRS.

Up-Rate

We first proceeded for each node by increasing 10 times (Fast) or decreasing 10 times (Slow) to mutate the Up-Rate.

Fast

In the figure below are reported the stacked bar charts of the probabilities for the CRS readout:

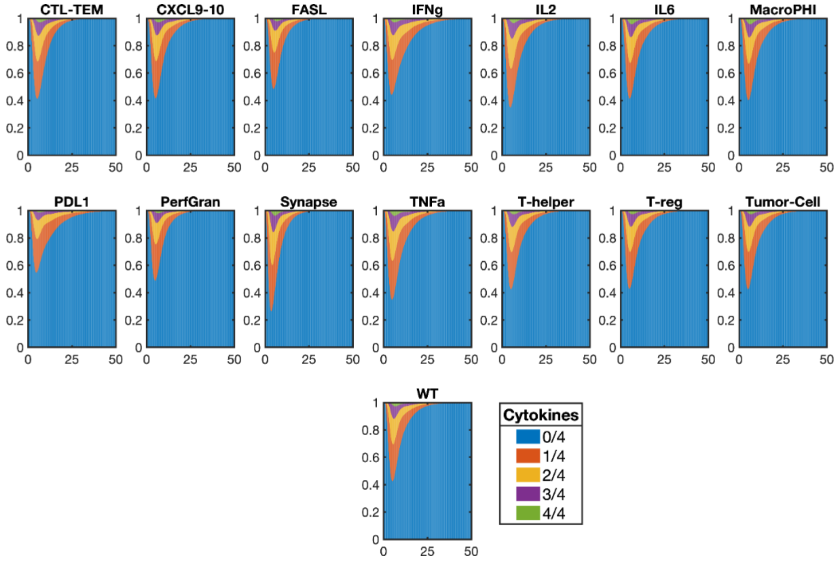

Figure S24: Stacked bar charts, representing the probabilities that the CRS variable has a specific value at every time step. Each chart represents a single perturbation in which the Up-Rate of the specified variable has been increased 10 times.

and then the average value by applying the formula (3):

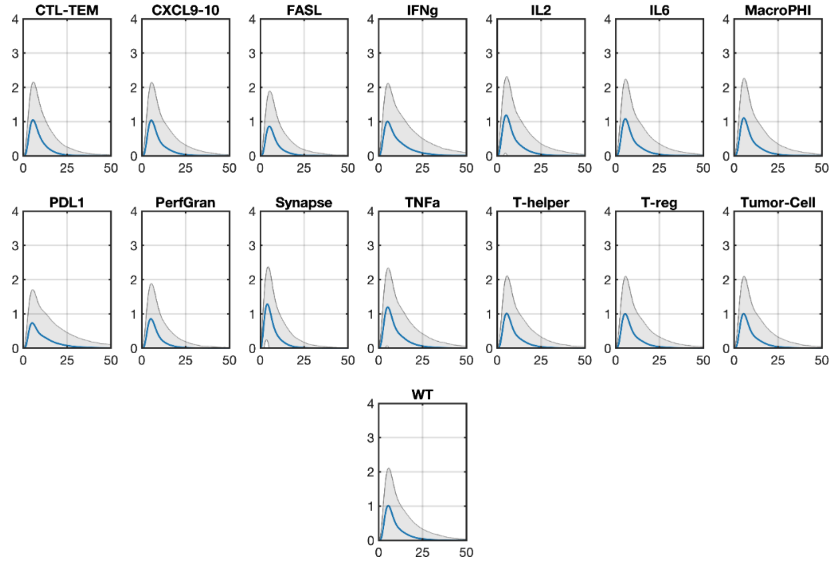

Figure S25: CRS average value. Each chart represents a single perturbation of the system, in this case increasing 10 times the Up-Rate. In blue are reported the averages while the shaded grey area shows plus/minus one standard deviation limited to the maximum and minimum values.

Slow

In the figure below are reported the stacked-bar charts of the probabilities for the CRS readout:

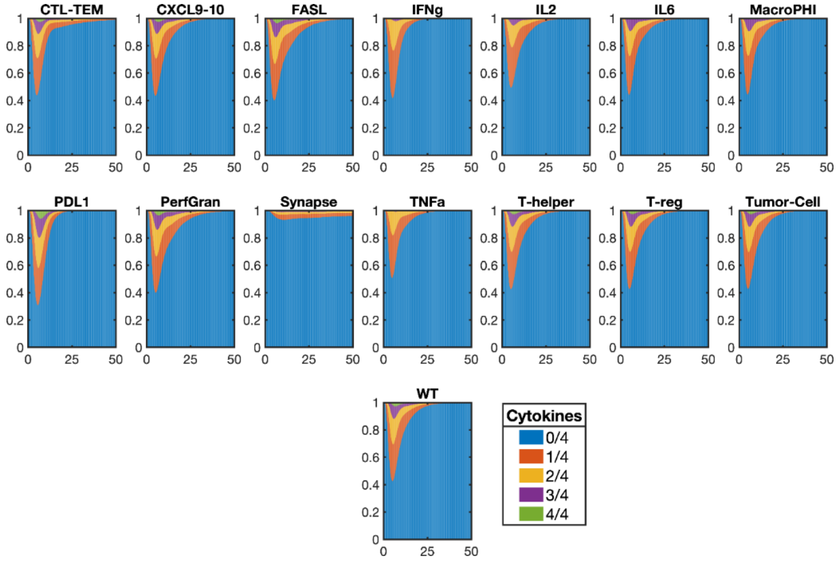

Figure S26: Stacked bar charts, representing the probabilities that the CRS variable has a specific value at every time step. Each chart represents a single perturbation in which the Up-Rate of the specified variable has been decreased 10 times.

and then the average value by applying the formula (3):

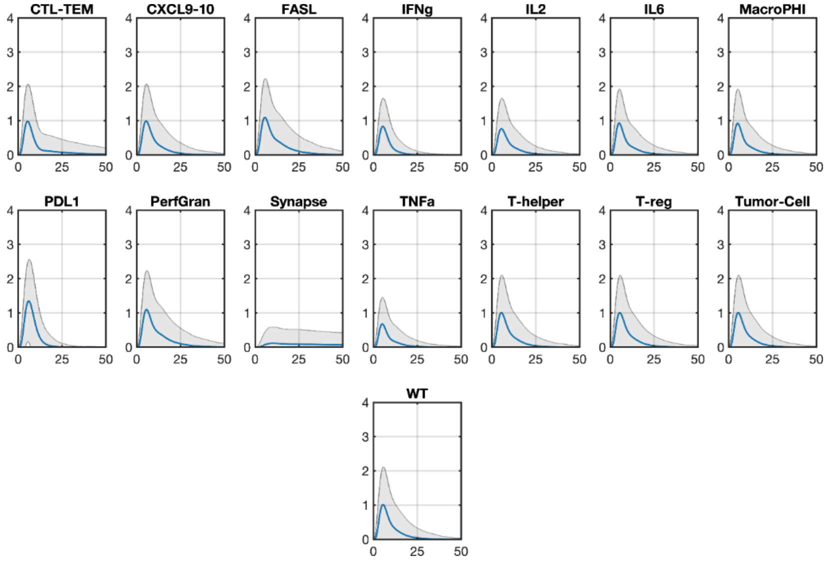

Figure S27: CRS average value. Each chart represents a single perturbation of the system, in this case decreasing 10 times the Up-Rate. In blue are reported the averages while the shaded grey area shows plus/minus one standard deviation limited to the maximum and minimum values.

Down-Rate

Fast

In the figure below are reported the stacked-bar charts of the probabilities for the CRS readout:

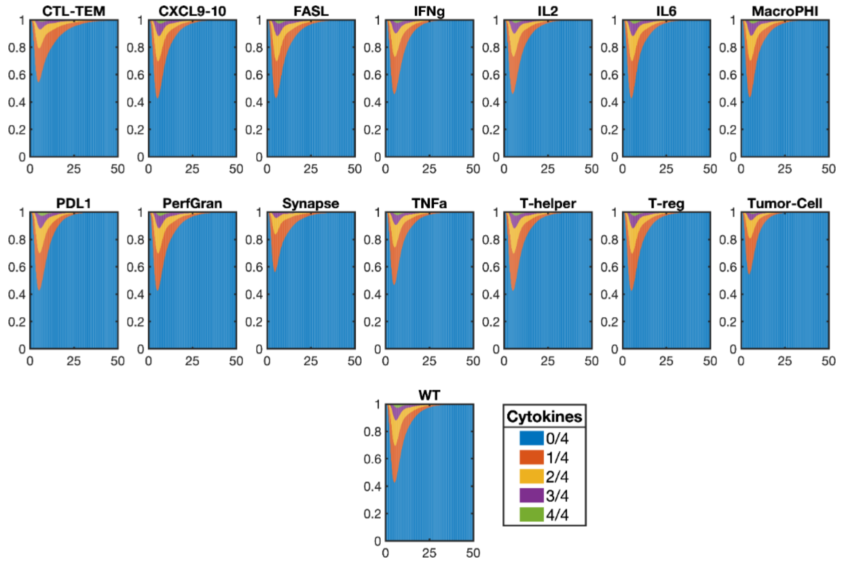

Figure S28: Stacked bar charts, representing the probabilities that the CRS variable has a specific value at every time step. Each chart represents a single perturbation in which the Up-Rate of the specified variable has been decreased 10 times.

and then the average value by applying the formula (3):

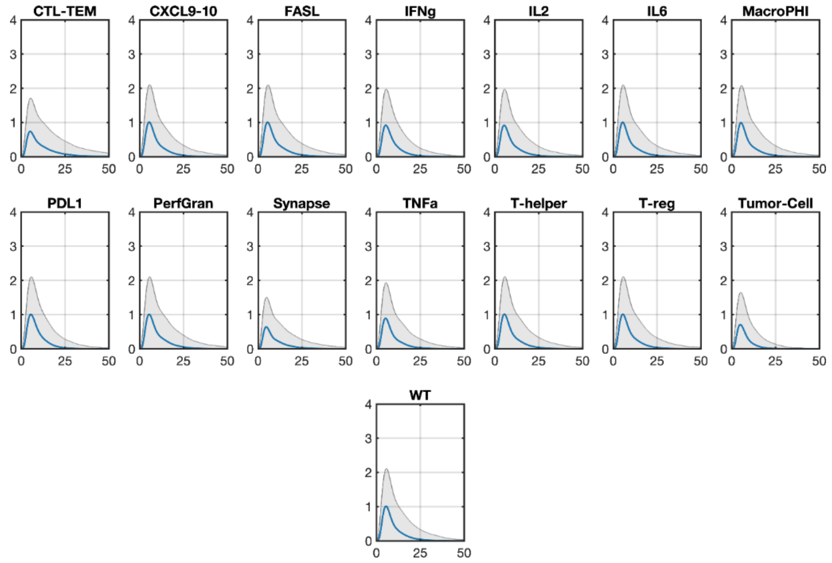

Figure S29: CRS average value. Each chart represents a single perturbation of the system, in this case increasing 10 times the Down-Rate. In blue are reported the averages while the shaded grey area shows plus/minus one standard deviation limited to the maximum and minimum values.

Slow

In the figure below are reported the stacked bar charts of the probabilities for the CRS readout:

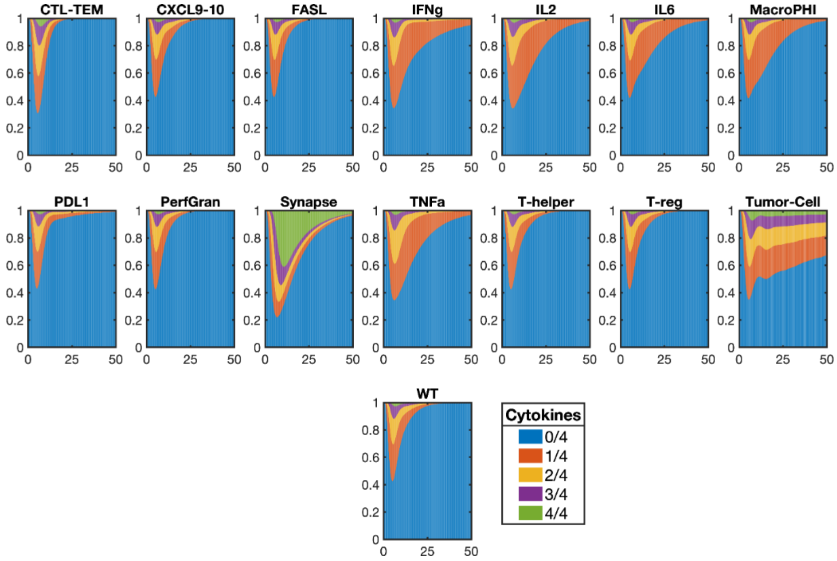

Figure S30: Stacked bar charts, representing the probabilities that the CRS variable has a specific value at every time step. Each chart represents a single perturbation in which the Down-Rate of the specified variable has been decreased 10 times.

and then the average value by applying the formula (3):

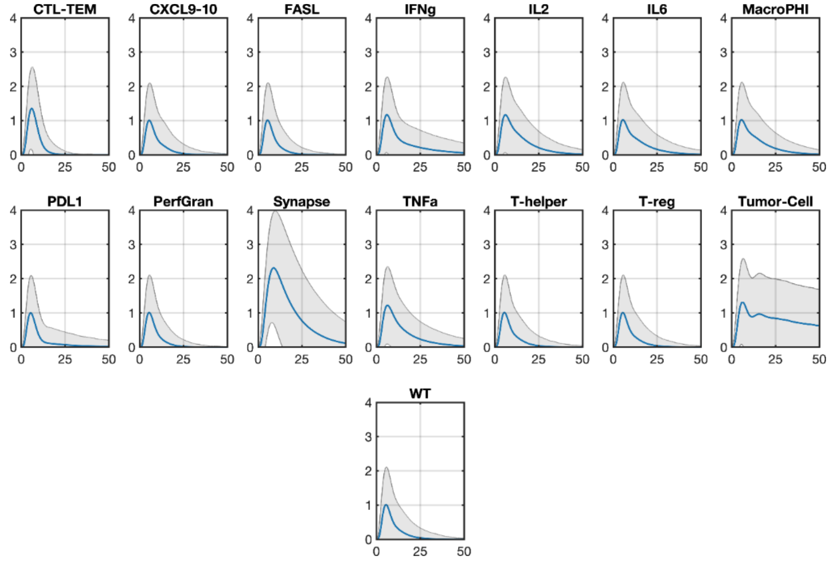

Figure S31: CRS average value. Each chart represents a single perturbation of the system, in this case decreasing 10 times the Down-Rate. In blue are reported the averages while the shaded grey area shows plus/minus one standard deviation limited to the maximum and minimum values.

CRS sensitivities to update rate perturbations

The average values obtained are then compared with the wild type behavior, to calculate the sensitivities accordingly to the equation (1).

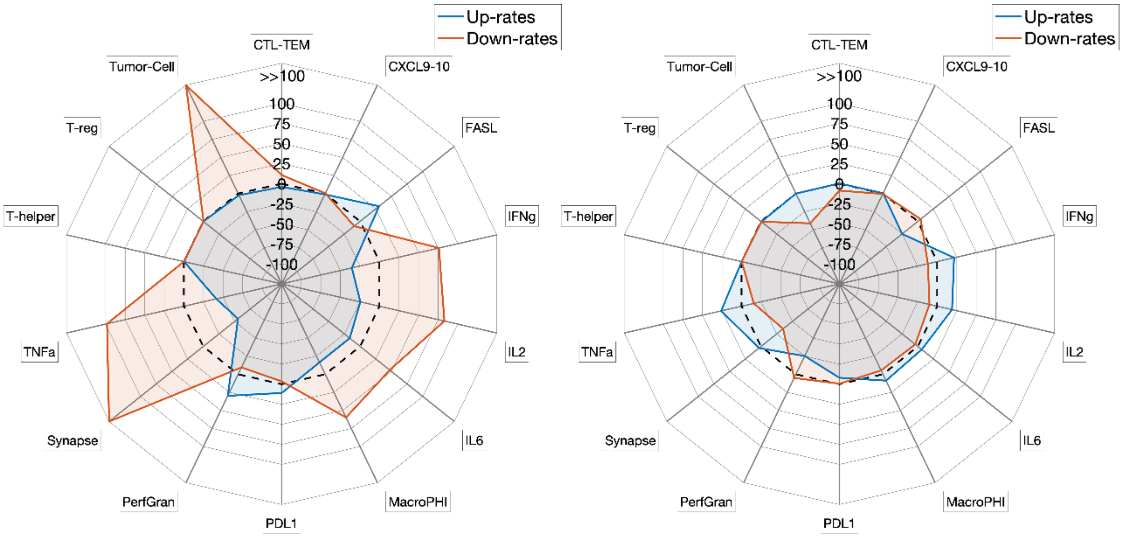

Figure S32: Sensitivity of the CRS when mutating the rates of increase (Up-rate) or decrease (Down-rate) of a variable. (Right) the rates are decreased 10 times compared to the wild type model, (left) they are increased 10 times.

Delayed combined therapy analysis

To study the effects of a delayed combined therapy with other drugs on the CRS and the tumor clearance, we performed a series of simulations in which the knock-down effect is put in action after the beginning of the BiAbs therapy.

Anti-PDL1 therapy

We performed the simulations starting from a tumor initial condition and at t = 1, 2.5, 5 and 7 [a.u.] introduced the PDL1-KD100. However, this does not imply an immediate elimination of PDL1 but rather a progressive process in which the variable tends to 0.

In the figure below are reported the stacked bar charts of the probabilities for the Tumor readout:

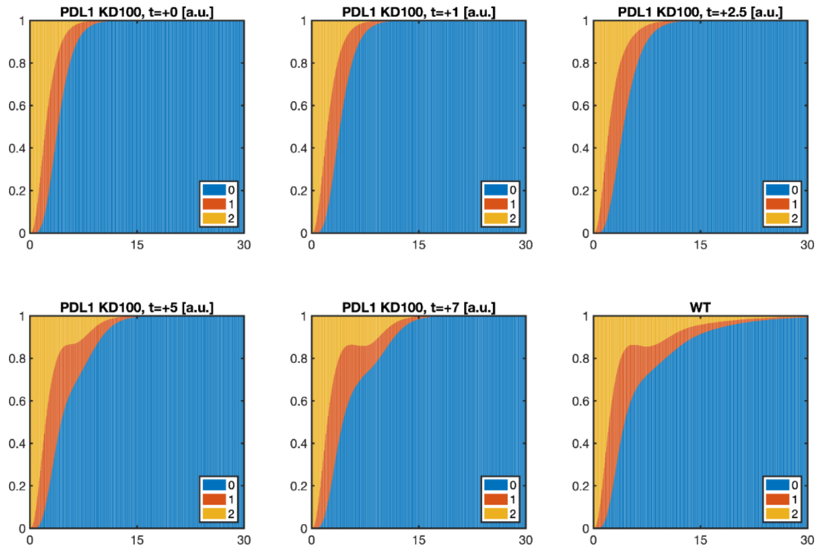

Figure S33: Stacked bar charts, representing the probabilities that the Tumor variable has a specific value at every time step. Each chart represents the effect of starting a combined therapy with anti-PDL1 at the specified time.

and then by applying the formula (2):

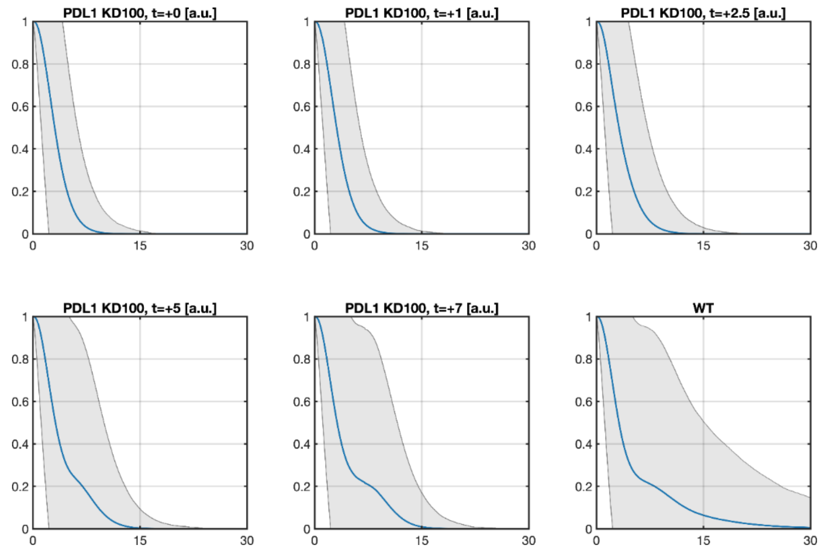

Figure S34: Tumor average value. Each chart represents the effect of starting a combined therapy with anti-PDL1 at the specified time. In blue are reported the averages while the shaded grey area shows plus/minus one standard deviation limited to the maximum and minimum values.

In the figure below are reported the stacked bar charts of the probabilities for the CRS readout:

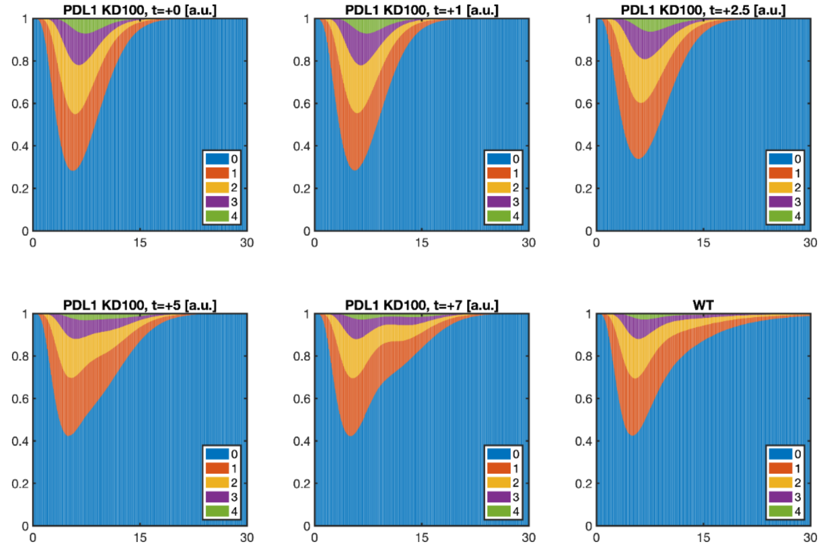

Figure S35: Stacked bar charts, representing the probabilities that the CRS variable has a specific value at every time step. Each chart represents the effect of starting a combined therapy with anti-PDL1 at the specified time.

and then by applying formula (3):

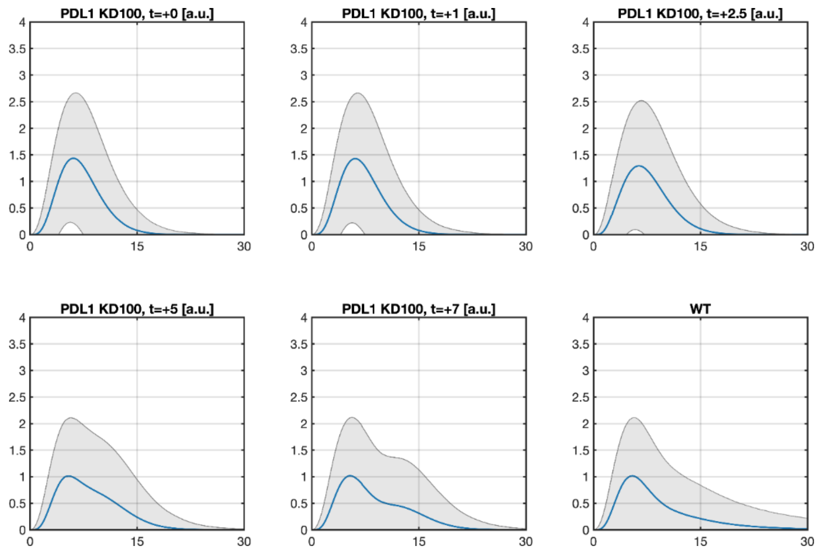

Figure S36: CRS average value. Each chart represents the effect of starting a combined therapy with anti-PDL1 at the specified time. In blue are reported the averages while the shaded grey area shows plus/minus one standard deviation limited to the maximum and minimum values.

Anti-TNFα therapy

TNFα-KD50

We performed the simulations starting from a tumor initial condition and at t = 1, 2.5, 5 and 7 [a.u.] introduced the TNFα-KD50. The variable has now an upper-bound that is 50% of its maximum value and is then progressively reduced until the limit is reached.

In the figure below are reported the stacked bar charts of the probabilities for the Tumor readout:

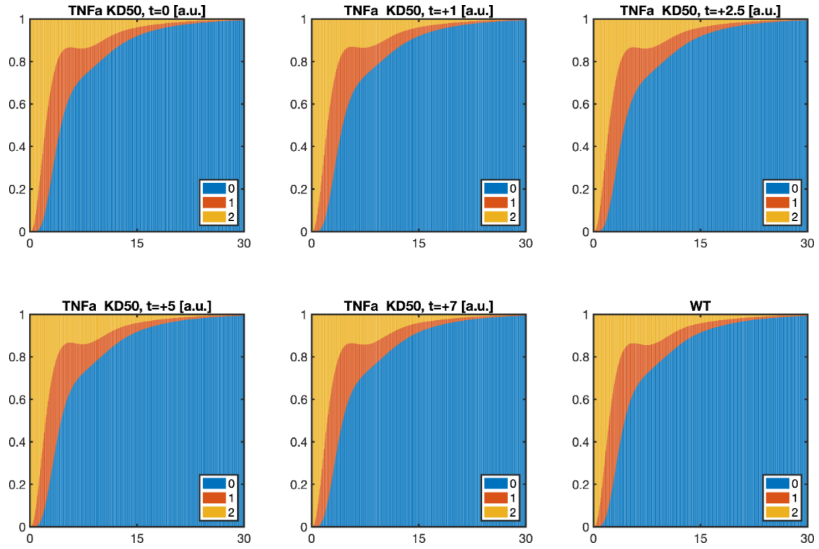

Figure S37: Stacked bar charts, representing the probabilities that the Tumor variable has a specific value at every time step. Each chart represents the effect of starting a combined therapy with anti-TNFα (KD50) at the specified time.

and then by applying the formula (2):

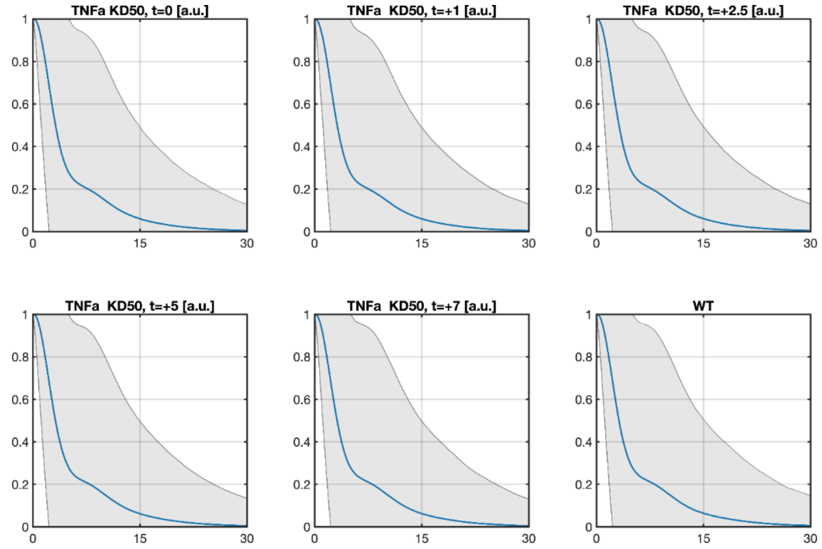

Figure S38: Tumor average value. Each chart represents the effect of starting a combined therapy with anti-TNFα (KD50) at the specified time. In blue are reported the averages while the shaded grey area shows plus/minus one standard deviation limited to the maximum and minimum values.

In the figure below are reported the stacked-bar charts of the probabilities for the CRS readout:

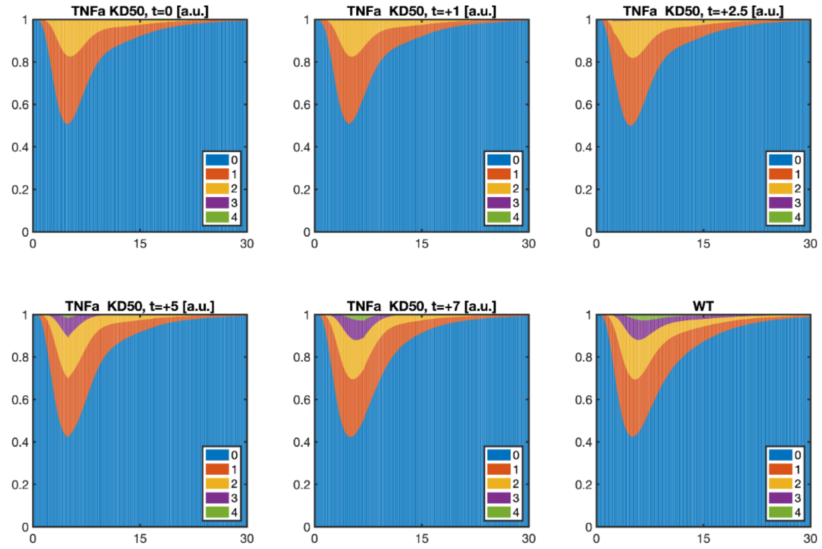

Figure S39: Stacked bar charts, representing the probabilities that the CRS variable has a specific value at every time step. Each chart represents the effect of starting a combined therapy with anti-TNFα (KD50) at the specified time.

and then by applying the formula (3):

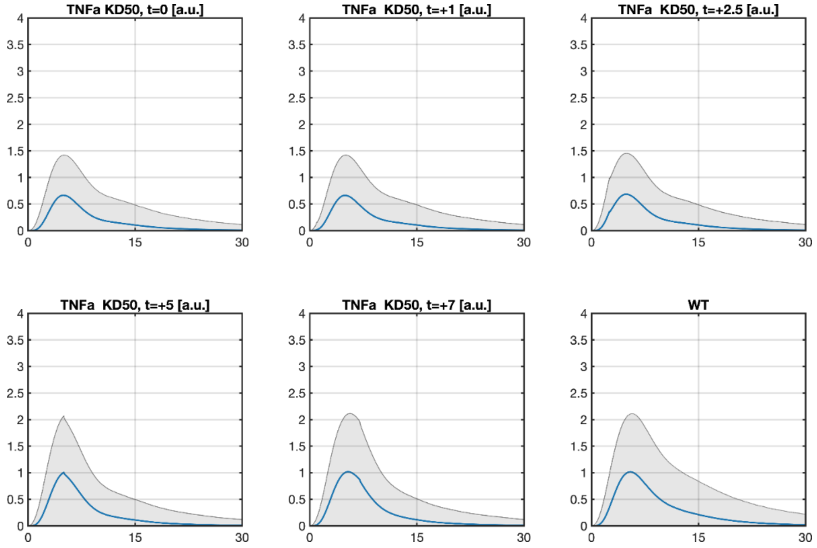

Figure S40: CRS average value. Each chart represents the effect of starting a combined therapy with anti-TNFα (KD50) at the specified time. In blue are reported the averages while the shaded grey area shows plus/minus one standard deviation limited to the maximum and minimum values.

The comparison between the averages of the different conditions are reported below:

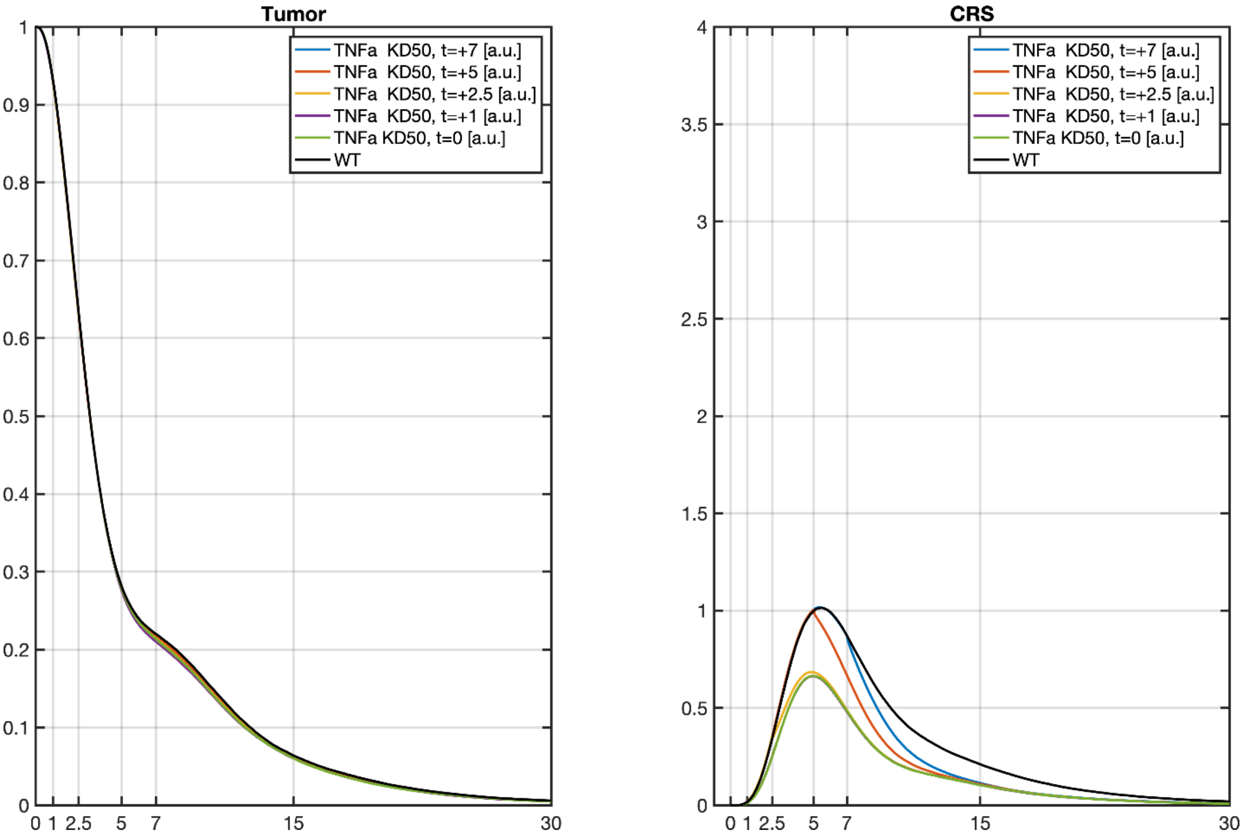

Figure S41: (left) Tumor clearance (right) CRS response after a continuous BiAbs treatment (high dose) starting at t=0 (WT condition). The different lines represent Tumor and CRS in the condition in which together with the BiAbs an anti-TNFα (KD50) is administered at time t = +1,+2.5,+5, and + 7 [a.u.]

TNFα-KD100

We perform the simulations starting from a tumor initial condition and at t = 1, 2.5, 5 and 7 [a.u.] introduced the TNFα-KD100, however this doesn't imply an immediate elimination of TNFα but rather a progressive process in which the variable tends to 0.

In the figure below are reported the stacked bar charts of the probabilities for the Tumor readout:

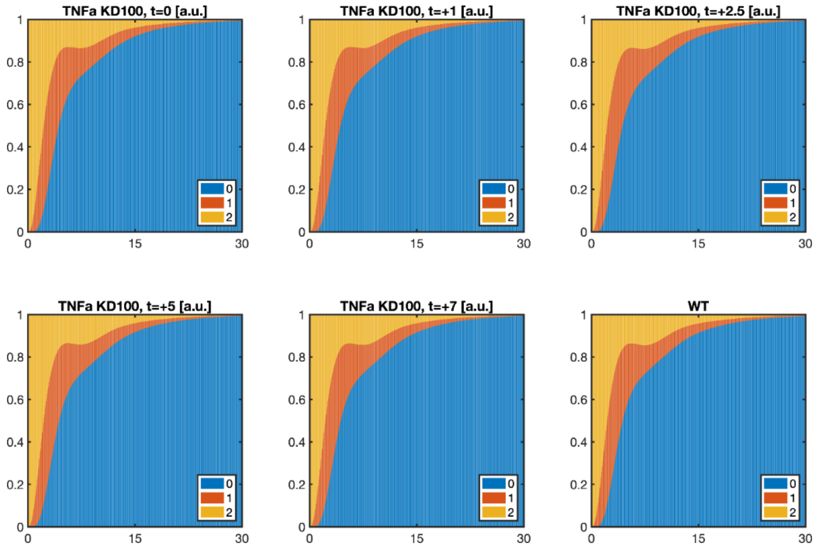

Figure S42: Stacked bar charts, representing the probabilities that the Tumor variable has a specific value at every time step. Each chart represents the effect of starting a combined therapy with anti-TNFα (KD100) at the specified time.

and then by applying the formula (2):

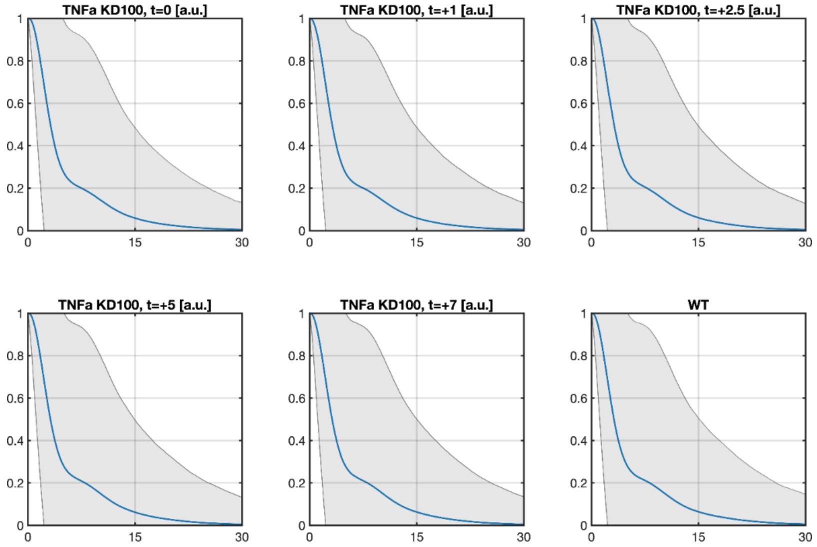

Figure S43: Tumor average value. Each chart represents the effect of starting a combined therapy with anti-TNFα (KD100) at the specified time. In blue are reported the averages while the shaded grey area shows plus/minus one standard deviation limited to the maximum and minimum values.

In the figure below are reported the stacked bar charts of the probabilities for the CRS readout:

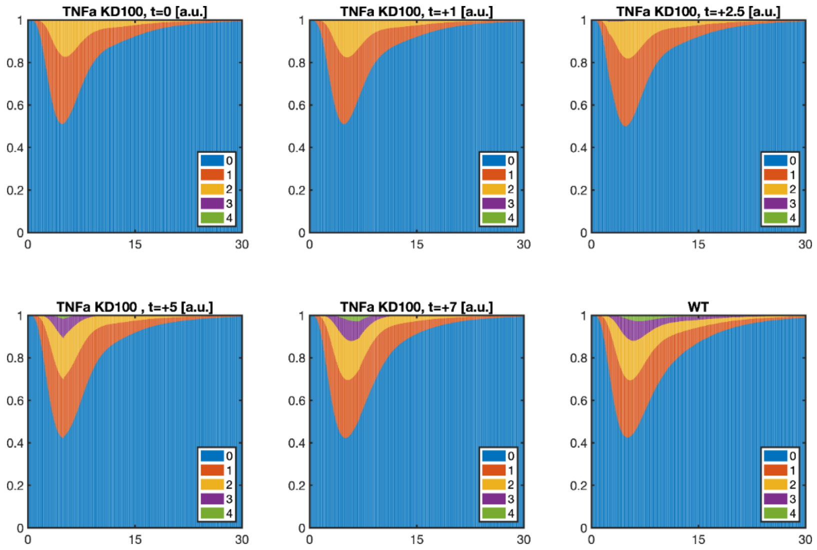

Figure S44: Stacked bar charts, representing the probabilities that the CRS variable has a specific value at every time step. Each chart represents the effect of starting a combined therapy with anti-TNFα (KD100) at the specified time.

and then by applying the formula (3):

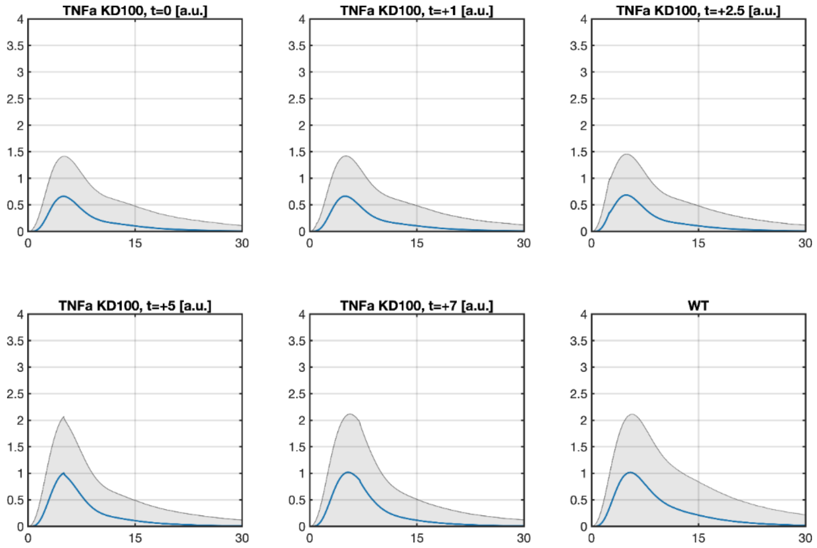

Figure S45: CRS average value. Each chart represents the effect of starting a combined therapy with anti-TNFα (KD100) at the specified time. In blue are reported the averages while the shaded grey area shows plus/minus one standard deviation limited to the maximum and minimum values.

References

1. Suryadevara CM, Gedeon PC, Sanchez-Perez L, Verla T, Alvarez-Breckenridge C, Choi BD, et al. Are BiTEs the “missing link” in cancer therapy? [Internet]. Oncoimmunology. Taylor and Francis Inc.; 2015 [cited 2020 Mar 16]. page e1008339. Available from: http://www.ncbi.nlm.nih.gov/pubmed/26155413
2. Aigner M, Feulner J, Schaffer S, Kischel R, Kufer P, Schneider K, et al. T lymphocytes can be effectively recruited for ex vivo and in vivo lysis of AML blasts by a novel CD33/CD3-bispecific BiTE antibody construct. Leukemia [Internet]. 2013 [cited 2020 Mar 16];27:1107–15. Available from: http://www.ncbi.nlm.nih.gov/pubmed/23178753
3. Sharpe AH, Pauken KE. The diverse functions of the PD1 inhibitory pathway [Internet]. Nat. Rev. Immunol. Nature Publishing Group; 2018 [cited 2020 Mar 16]. page 153–67. Available from: http://www.ncbi.nlm.nih.gov/pubmed/28990585
4. Galon J, Bruni D. Approaches to treat immune hot, altered and cold tumours with combination immunotherapies [Internet]. Nat. Rev. Drug Discov. Nature Publishing Group; 2019 [cited 2020 Mar 16]. page 197–218. Available from: http://www.ncbi.nlm.nih.gov/pubmed/30610226
5. Wing JB, Tanaka A, Sakaguchi S. Human FOXP3 + Regulatory T Cell Heterogeneity and Function in Autoimmunity and Cancer [Internet]. Immunity. Cell Press; 2019 [cited 2020 Mar 16]. page 302–16. Available from: http://www.ncbi.nlm.nih.gov/pubmed/30784578
6. Spolski R, Li P, Leonard WJ. Biology and regulation of IL-2: from molecular mechanisms to human therapy. Nat. Rev. Immunol. Nature Publishing Group; 2018. page 648–59.
7. Castro F, Cardoso AP, Gonçalves RM, Serre K, Oliveira MJ. Interferon-gamma at the crossroads of tumor immune surveillance or evasion [Internet]. Front. Immunol. Frontiers Media S.A.; 2018 [cited 2020 Mar 16]. page 847. Available from: http://www.ncbi.nlm.nih.gov/pubmed/29780381
8. Voskoboinik I, Whisstock JC, Trapani JA. Perforin and granzymes: Function, dysfunction and human pathology [Internet]. Nat. Rev. Immunol. Nature Publishing Group; 2015 [cited 2020 Mar 16]. page 388–400. Available from: http://www.ncbi.nlm.nih.gov/pubmed/25998963
9. Martínez-Lostao L, Anel A, Pardo J. How Do Cytotoxic Lymphocytes Kill Cancer Cells? Clin Cancer Res [Internet]. American Association for Cancer Research Inc.; 2015 [cited 2020 Mar 16];21:5047–56. Available from: http://www.ncbi.nlm.nih.gov/pubmed/26567364
10. Fisher DT, Appenheimer MM, Evans SS. The two faces of IL-6 in the tumor microenvironment [Internet]. Semin. Immunol. Academic Press; 2014 [cited 2020 Mar 16]. page 38–47. Available from: http://www.ncbi.nlm.nih.gov/pubmed/24602448
11. Shimabukuro-Vornhagen A, Gödel P, Subklewe M, Stemmler HJ, Schlößer HA, Schlaak M, et al. Cytokine release syndrome [Internet]. J. Immunother. Cancer. BioMed Central Ltd.; 2018 [cited 2020 Mar 16]. page 56. Available from: http://www.ncbi.nlm.nih.gov/pubmed/29907163
12. Brudno JN, Kochenderfer JN. Toxicities of chimeric antigen receptor T cells: Recognition and management [Internet]. Blood. American Society of Hematology; 2016 [cited 2020 Mar 16]. page 3321–30. Available from: http://www.ncbi.nlm.nih.gov/pubmed/27207799
13. Liao W, Lin JX, Leonard WJ. Interleukin-2 at the Crossroads of Effector Responses, Tolerance, and Immunotherapy. Immunity. 2013. page 13–25.

14. Li J, Ybarra R, Mak J, Herault A, De Almeida P, Arrazate A, et al. IFNg-induced Chemokines Are Required for CXCR3-mediated T-Cell Recruitment and Antitumor Efficacy of Anti-HER2/CD3 Bispecific Antibody. Clin Cancer Res [Internet]. American Association for Cancer Research Inc.; 2018 [cited 2020 Mar 16];24:6447–58. Available from: http://www.ncbi.nlm.nih.gov/pubmed/29950350
15. Krupka C, Kufer P, Kischel R, Zugmaier G, Lichtenegger FS, Köhnke T, et al. Blockade of the PD-1/PD-L1 axis augments lysis of AML cells by the CD33/CD3 BiTE antibody construct AMG 330: Reversing a T-cell-induced immune escape mechanism. Leukemia [Internet]. Nature Publishing Group; 2016 [cited 2020 Mar 16];30:484–91. Available from: http://www.ncbi.nlm.nih.gov/pubmed/26239198
16. Li J, Piskol R, Ybarra R, Chen YJJ, Li J, Slaga D, et al. CD3 bispecific antibody-induced cytokine release is dispensable for cytotoxic T cell activity. Sci Transl Med. American Association for the Advancement of Science; 2019;11.
17. Giavridis T, Van Der Stegen SJC, Eyquem J, Hamieh M, Piersigilli A, Sadelain M. CAR T cell-induced cytokine release syndrome is mediated by macrophages and abated by IL-1 blockade letter. Nat Med [Internet]. Nature Publishing Group; 2018 [cited 2020 Mar 16];24:731–8. Available from: http://dx.doi.org/10.1038/s41591-018-0041-7
18. Norelli M, Camisa B, Barbiera G, Falcone L, Purevdorj A, Genua M, et al. Monocyte-derived IL-1 and IL-6 are differentially required for cytokine-release syndrome and neurotoxicity due to CAR T cells. Nat Med. Nature Publishing Group; 2018;24:739–48.
19. Teachey DT, Lacey SF, Shaw PA, Melenhorst JJ, Maude SL, Frey N, et al. Identification of Predictive Biomarkers for Cytokine Release Syndrome after Chimeric Antigen Receptor T-cell Therapy for Acute Lymphoblastic Leukemia. Cancer Discov [Internet]. Cancer Discov; 2016 [cited 2022 Jan 12];6:664–79. Available from: https://pubmed.ncbi.nlm.nih.gov/27076371/
